# Supplementary material for: ZMYM2 controls human transposable element transcription through distinct co-regulatory complexes
Source: eLife. 2023 Nov 7;12:RP86669. doi: 10.7554/eLife.86669 (PMC10629813; doi:10.7554/eLife.86669)
Supplement: Figure 1—source data 1. — Resulting proteins were detected by immunoblotting (IB) with ZMYM2 and ADNP antibodies. The regions used for creating the final figure are boxed. Molecular weight marker sizes (kDa) are shown on the left. [file elife-86669-fig1-data1.zip › Figure1Sourcedata1/Figure1Sourcedata1.pptx]

## Slide 1
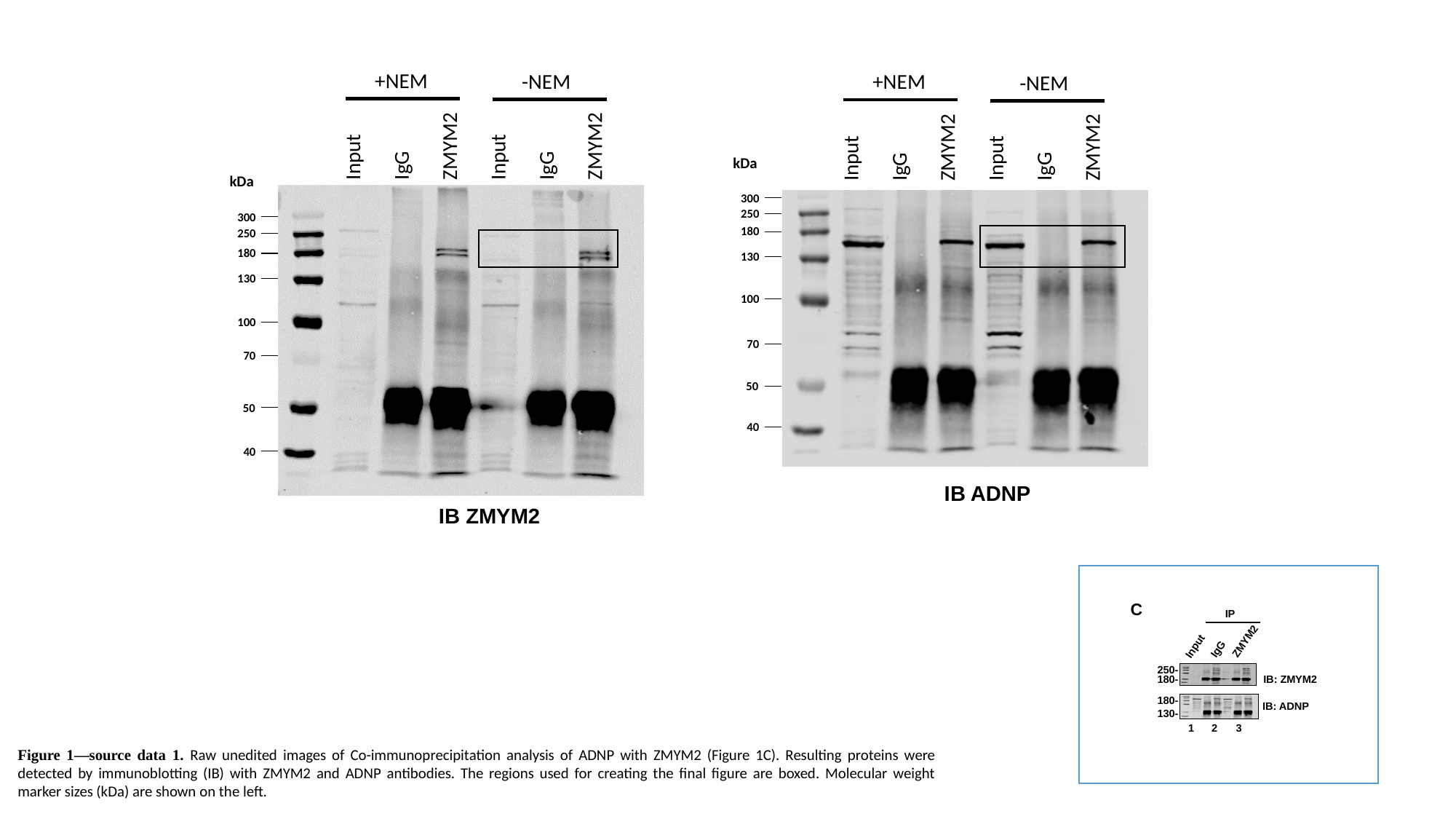

+NEM
-NEM
+NEM
-NEM
 Input
 Input
 ZMYM2
 ZMYM2
 ZMYM2
 ZMYM2
 IgG
 Input
 IgG
 Input
 IgG
 IgG
kDa
kDa
300
250
300
180
250
180
130
130
100
100
70
70
50
50
40
40
IB ADNP
IB ZMYM2
C
IP
 ZMYM2
 Input
 IgG
250-
180-
IB: ZMYM2
180-
IB: ADNP
130-
1
2
3
Figure 1—source data 1. Raw unedited images of Co-immunoprecipitation analysis of ADNP with ZMYM2 (Figure 1C). Resulting proteins were detected by immunoblotting (IB) with ZMYM2 and ADNP antibodies. The regions used for creating the final figure are boxed. Molecular weight marker sizes (kDa) are shown on the left.
