# Supplementary figures and images for: ZMYM2 controls human transposable element transcription through distinct co-regulatory complexes
Source: eLife. 2023 Nov 7;12:RP86669. doi: 10.7554/eLife.86669 (PMC10629813; doi:10.7554/eLife.86669)

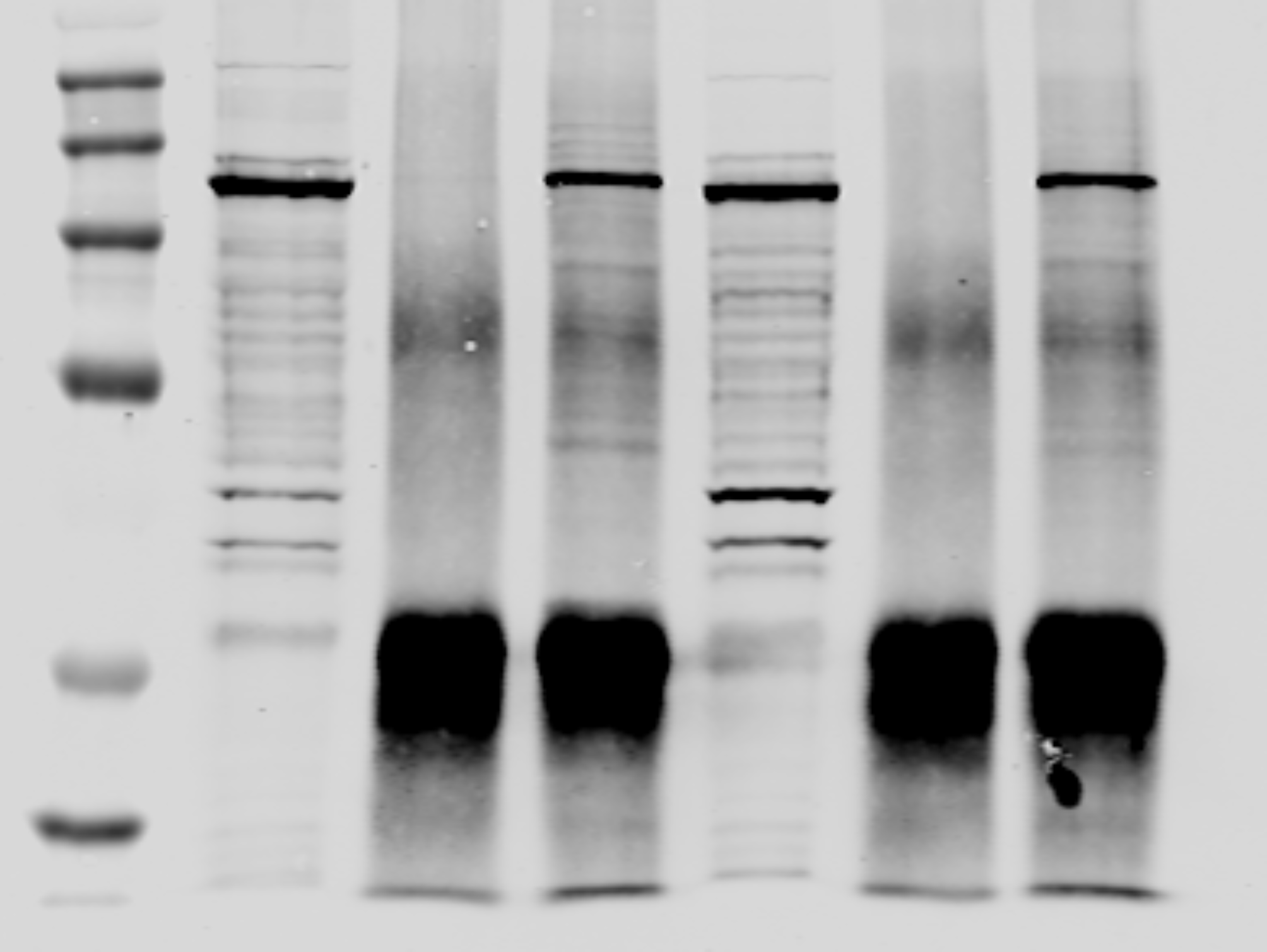

Supplement: Figure 1—source data 1. — Resulting proteins were detected by immunoblotting (IB) with ZMYM2 and ADNP antibodies. The regions used for creating the final figure are boxed. Molecular weight marker sizes (kDa) are shown on the left. [file elife-86669-fig1-data1.zip › Figure1Sourcedata1/Fig1C-source data aADNP.tif]

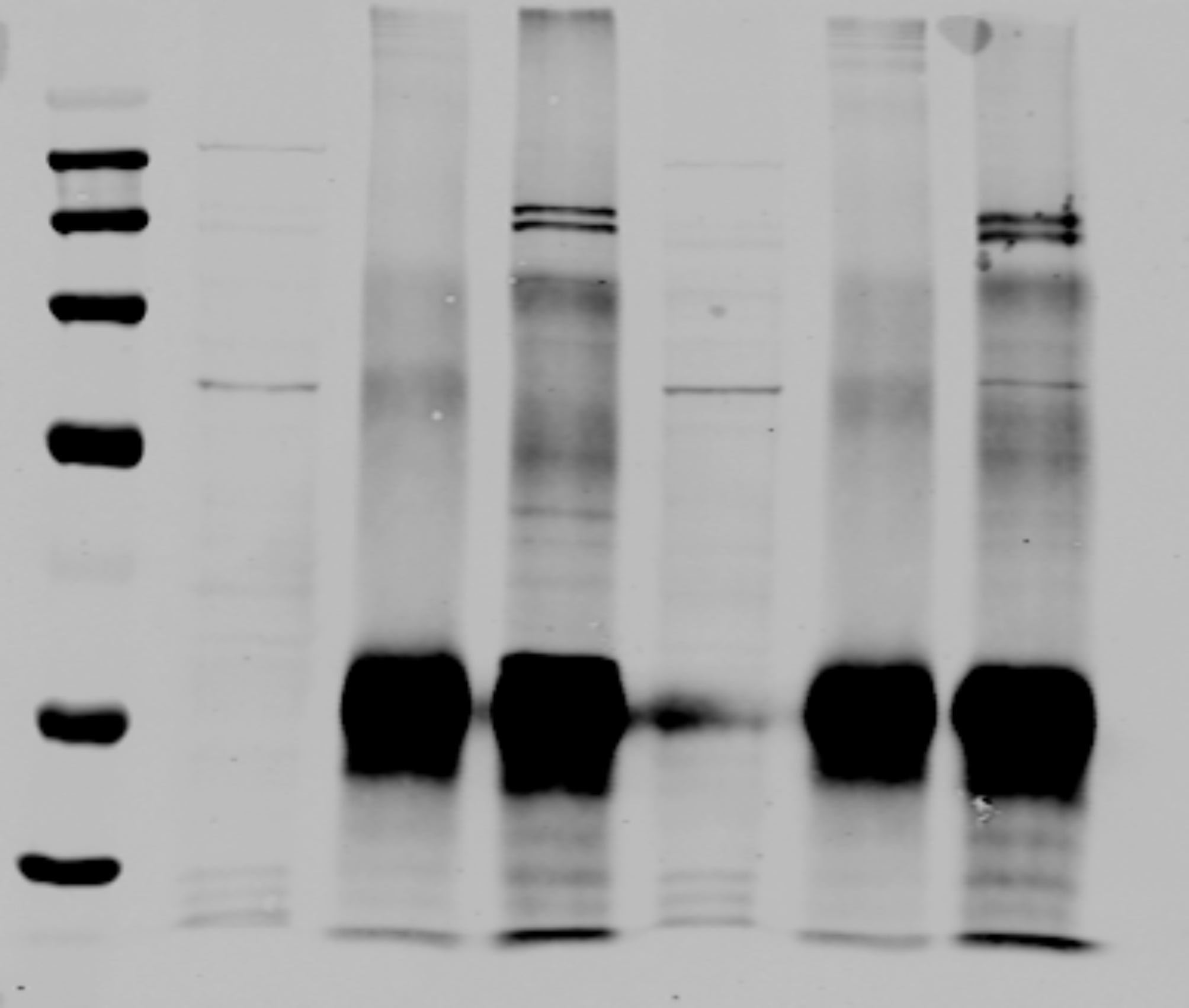

Supplement: Figure 1—source data 1. — Resulting proteins were detected by immunoblotting (IB) with ZMYM2 and ADNP antibodies. The regions used for creating the final figure are boxed. Molecular weight marker sizes (kDa) are shown on the left. [file elife-86669-fig1-data1.zip › Figure1Sourcedata1/Fig1C-source data aZMYM2.tif]

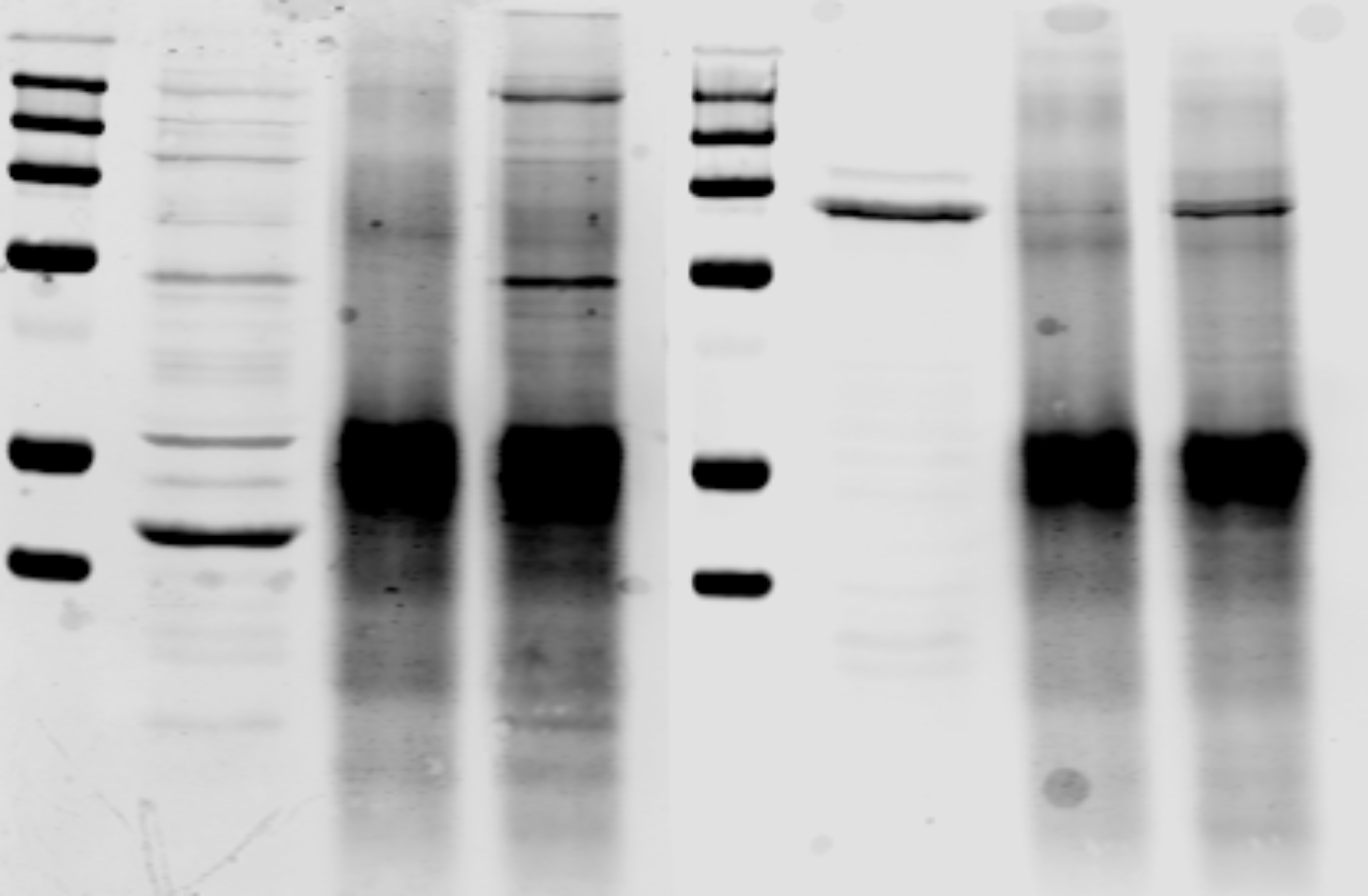

Supplement: Figure 1—figure supplement 1—source data 1. — Resulting proteins were detected by immunoblotting (IB) with ZMYM3 and TRIM28 antibodies. The regions used for creating the final figure are boxed. Molecular weight marker sizes (kDa) are shown on the left. [file elife-86669-fig1-figsupp1-data1.zip › Figure1S1Sourcedata1/FigS1B-souce data aZMYM3.tif]

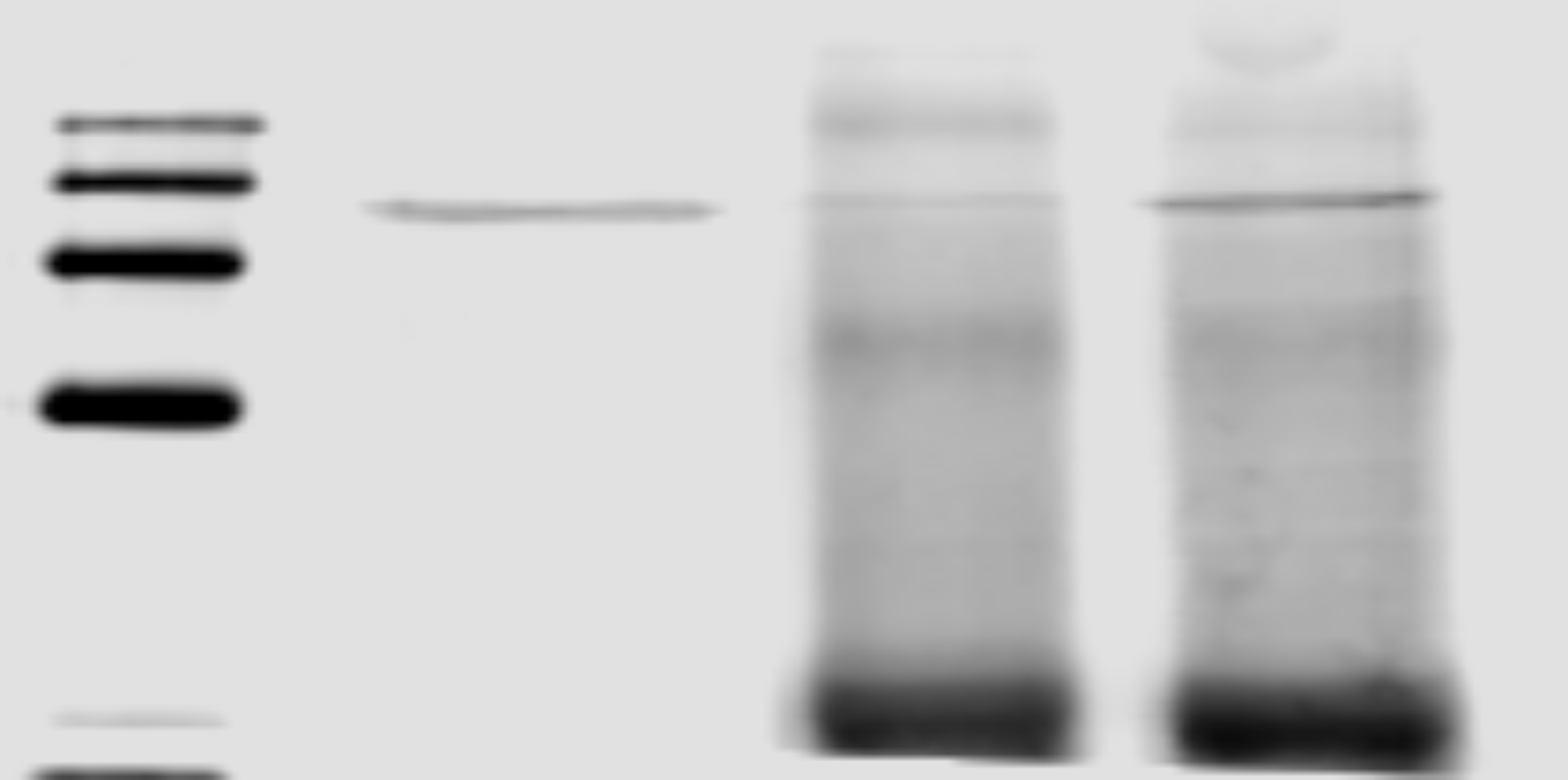

Supplement: Figure 1—figure supplement 1—source data 1. — Resulting proteins were detected by immunoblotting (IB) with ZMYM3 and TRIM28 antibodies. The regions used for creating the final figure are boxed. Molecular weight marker sizes (kDa) are shown on the left. [file elife-86669-fig1-figsupp1-data1.zip › Figure1S1Sourcedata1/FigS1B-souce data aADNP.tif]

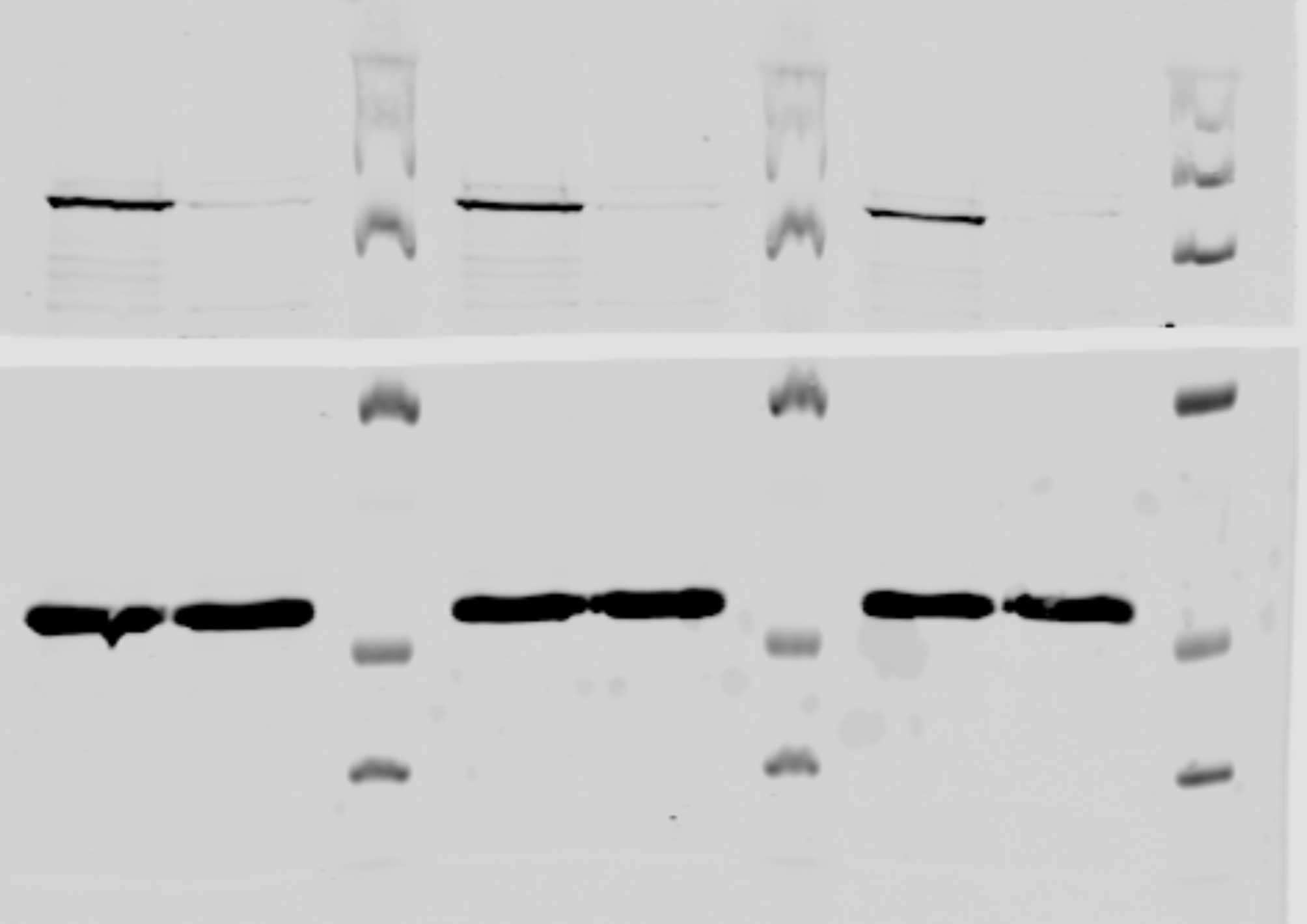

Supplement: Figure 1—figure supplement 1—source data 2. — αTubulin was used as a loading control (bottom). Molecular weight markers (M) are shown. The regions used for creating the final figure are boxed. Molecular weight marker sizes (kDa) are shown on the right. [file elife-86669-fig1-figsupp1-data2.zip › Figure1S1Sourcedata2/FigS1C-souce data aADNPaTUBLIN.tif]

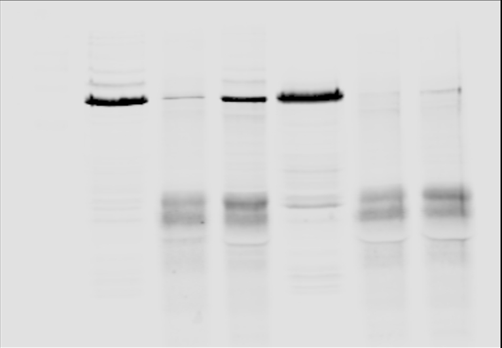

Supplement: Figure 3—figure supplement 1—source data 2. — Resulting proteins were detected by immunoblotting (IB) with SUMO2 or TRIM28 antibodies. The regions used for creating the final figure are boxed. Molecular weight marker sizes (kDa) are shown on the left. [file elife-86669-fig3-figsupp1-data2.zip › Figure3S1Sourcedata2/FigS3B-source data aTRIM28.tif]

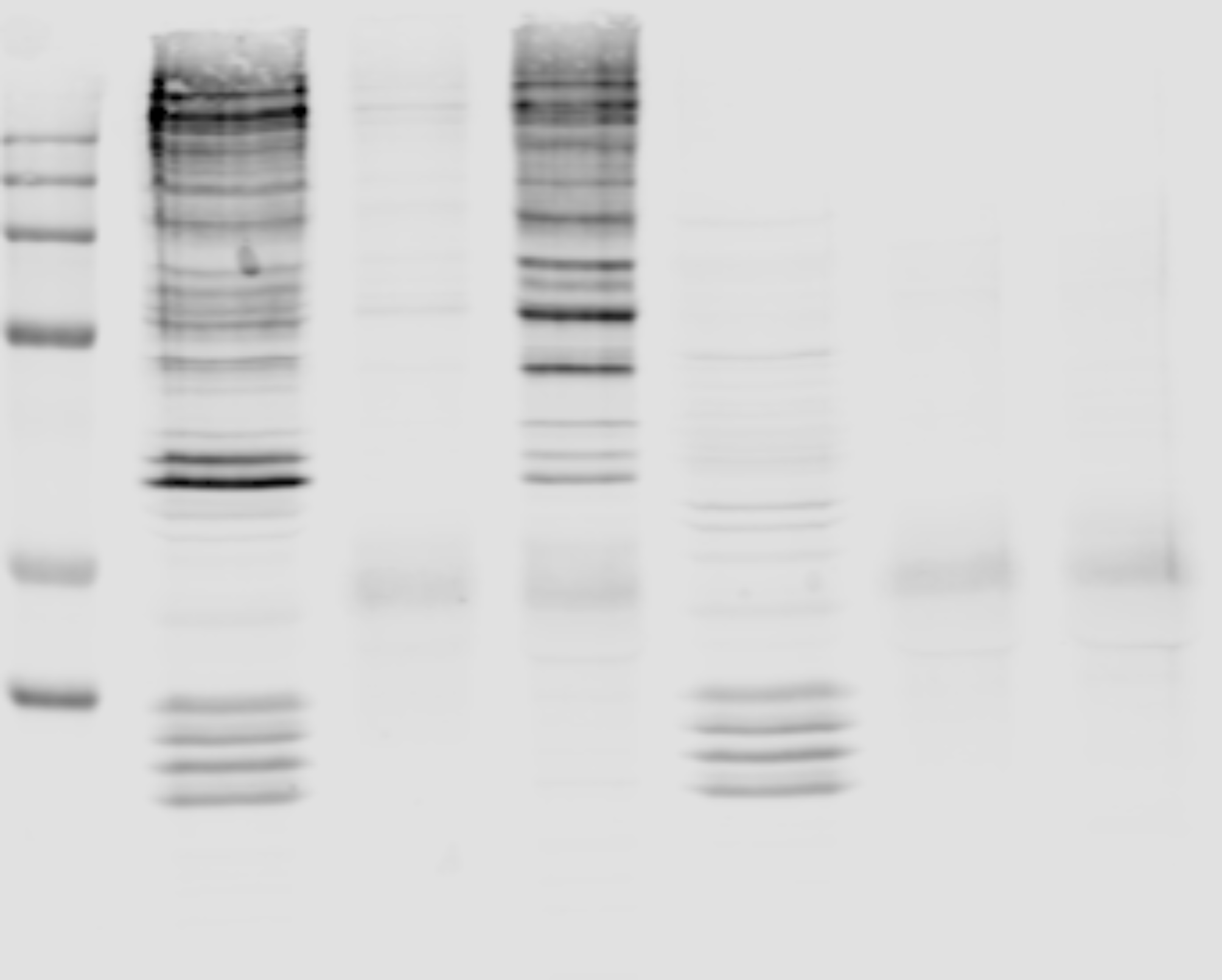

Supplement: Figure 3—figure supplement 1—source data 2. — Resulting proteins were detected by immunoblotting (IB) with SUMO2 or TRIM28 antibodies. The regions used for creating the final figure are boxed. Molecular weight marker sizes (kDa) are shown on the left. [file elife-86669-fig3-figsupp1-data2.zip › Figure3S1Sourcedata2/FigS3B-source data aSUMO2.tif]

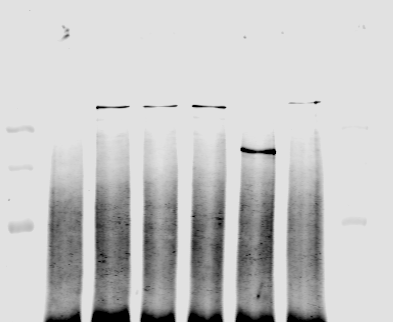

Supplement: Figure 3—figure supplement 1—source data 3. — IPs (top) were immunoblotted (IB) with the indicated antibodies. Resulting proteins were detected by immunoblotting (IB) with GFP or TRIM28 antibodies. The regions used for creating the final figure are boxed. Molecular weight marker sizes (kDa) are shown on the left. [file elife-86669-fig3-figsupp1-data3.zip › Figure3S1Sourcedata3/FigS3C-source data aGFP.tif]

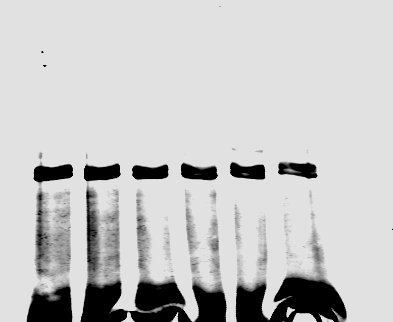

Supplement: Figure 3—figure supplement 1—source data 3. — IPs (top) were immunoblotted (IB) with the indicated antibodies. Resulting proteins were detected by immunoblotting (IB) with GFP or TRIM28 antibodies. The regions used for creating the final figure are boxed. Molecular weight marker sizes (kDa) are shown on the left. [file elife-86669-fig3-figsupp1-data3.zip › Figure3S1Sourcedata3/FigS3C-source data aTRIM28.tif]

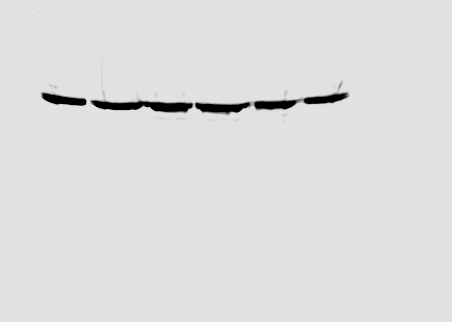

Supplement: Figure 3—figure supplement 1—source data 4. — Input samples were immunoblotted (IB) with the indicated antibodies. The regions used for creating the final figure are boxed. [file elife-86669-fig3-figsupp1-data4.zip › Figure3S1Sourcedata4/FigS3C-source data input aTRIM28.tif]

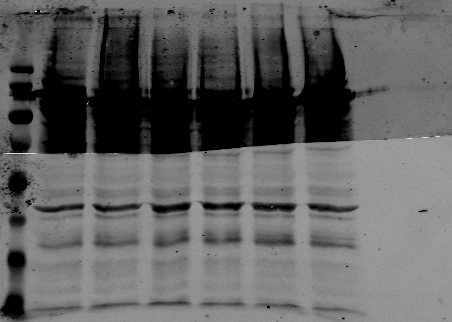

Supplement: Figure 3—figure supplement 1—source data 4. — Input samples were immunoblotted (IB) with the indicated antibodies. The regions used for creating the final figure are boxed. [file elife-86669-fig3-figsupp1-data4.zip › Figure3S1Sourcedata4/FigS3C-source data input aActin.tif]

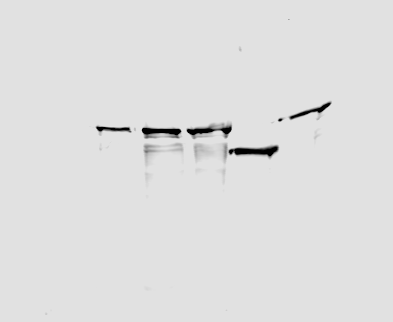

Supplement: Figure 3—figure supplement 1—source data 4. — Input samples were immunoblotted (IB) with the indicated antibodies. The regions used for creating the final figure are boxed. [file elife-86669-fig3-figsupp1-data4.zip › Figure3S1Sourcedata4/FigS3C-source data input aGFP.tif]

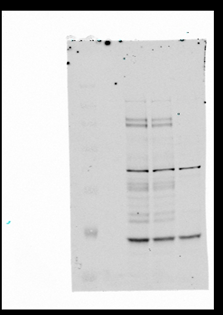

Supplement: Figure 3—figure supplement 1—source data 6. — Lamin B (loading control), TRIM28 and ZMYM2 were detected by immunoblotting (IB) (Figure 3—figure supplement 1G). The regions used for creating the final figure are boxed. Molecular weight marker sizes (kDa) are shown on the left. [file elife-86669-fig3-figsupp1-data6.zip › Figure3S1Sourcedata6/FigS3G-source data aZMYM2.tif]

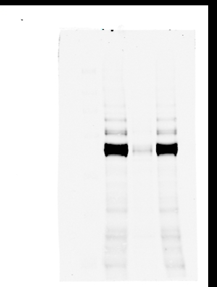

Supplement: Figure 3—figure supplement 1—source data 6. — Lamin B (loading control), TRIM28 and ZMYM2 were detected by immunoblotting (IB) (Figure 3—figure supplement 1G). The regions used for creating the final figure are boxed. Molecular weight marker sizes (kDa) are shown on the left. [file elife-86669-fig3-figsupp1-data6.zip › Figure3S1Sourcedata6/FigS3G-source data aTRIM28.tif]

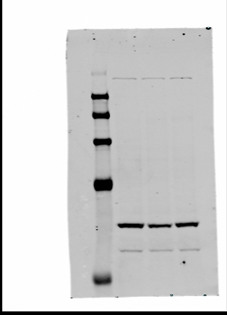

Supplement: Figure 3—figure supplement 1—source data 6. — Lamin B (loading control), TRIM28 and ZMYM2 were detected by immunoblotting (IB) (Figure 3—figure supplement 1G). The regions used for creating the final figure are boxed. Molecular weight marker sizes (kDa) are shown on the left. [file elife-86669-fig3-figsupp1-data6.zip › Figure3S1Sourcedata6/FigS3G-source data aLaminB.tif]

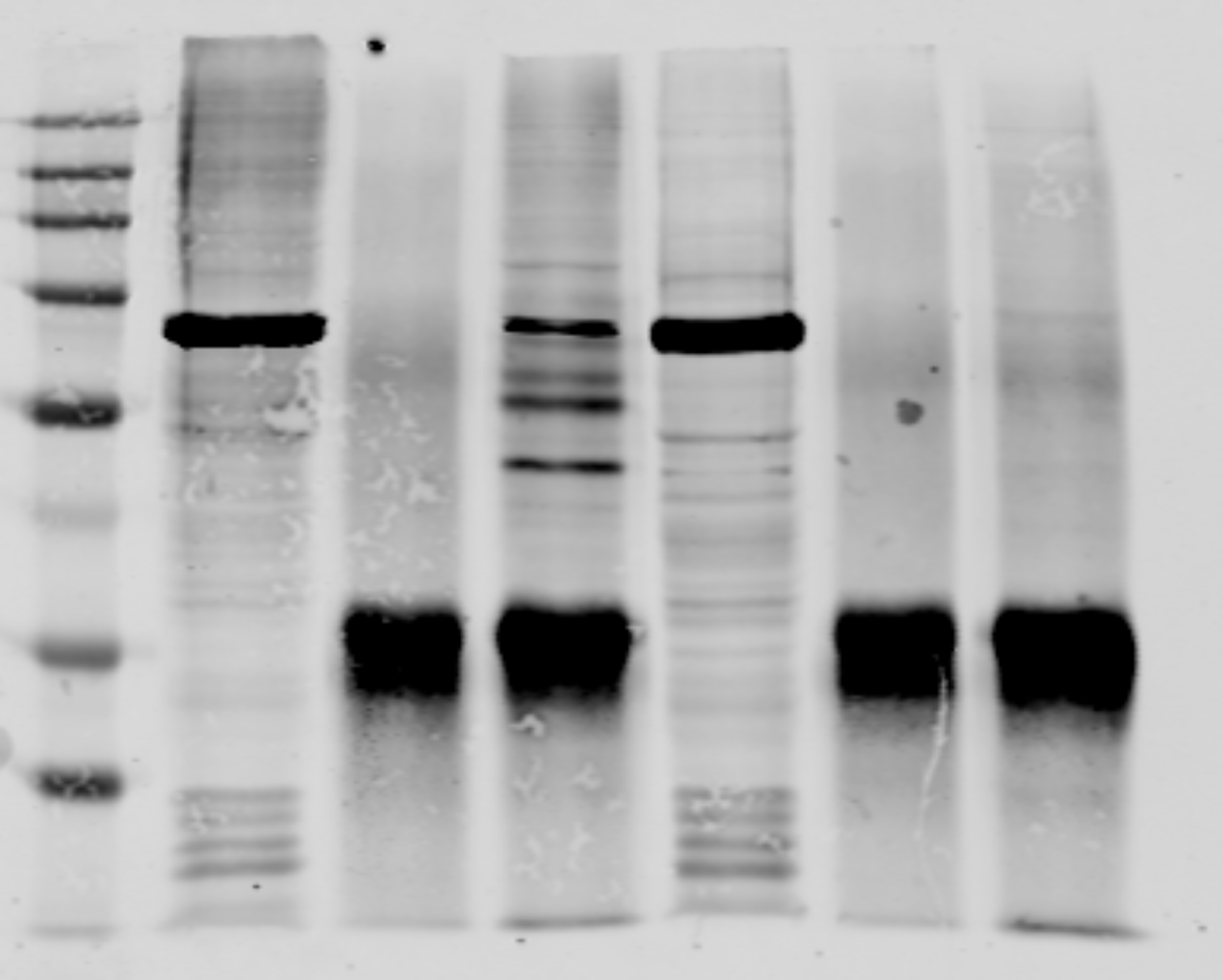

Supplement: Figure 4—source data 1. — Resulting proteins were detected by immunoblotting (IB) with ZMYM2 and TRIM28 antibodies. The regions used for creating the final figure are boxed. Molecular weight marker sizes (kDa) are shown on the left. [file elife-86669-fig4-data1.zip › Figure4Sourcedata1/Fig4A-source data aTRIM28.tif]
