# Supplementary material for: ZMYM2 controls human transposable element transcription through distinct co-regulatory complexes
Source: eLife. 2023 Nov 7;12:RP86669. doi: 10.7554/eLife.86669 (PMC10629813; doi:10.7554/eLife.86669)
Supplement: Figure 1—figure supplement 1—source data 1. — Resulting proteins were detected by immunoblotting (IB) with ZMYM3 and TRIM28 antibodies. The regions used for creating the final figure are boxed. Molecular weight marker sizes (kDa) are shown on the left. [file elife-86669-fig1-figsupp1-data1.zip › Figure1S1Sourcedata1/Figure1S1Sourcedata1.pptx]

## Slide 1
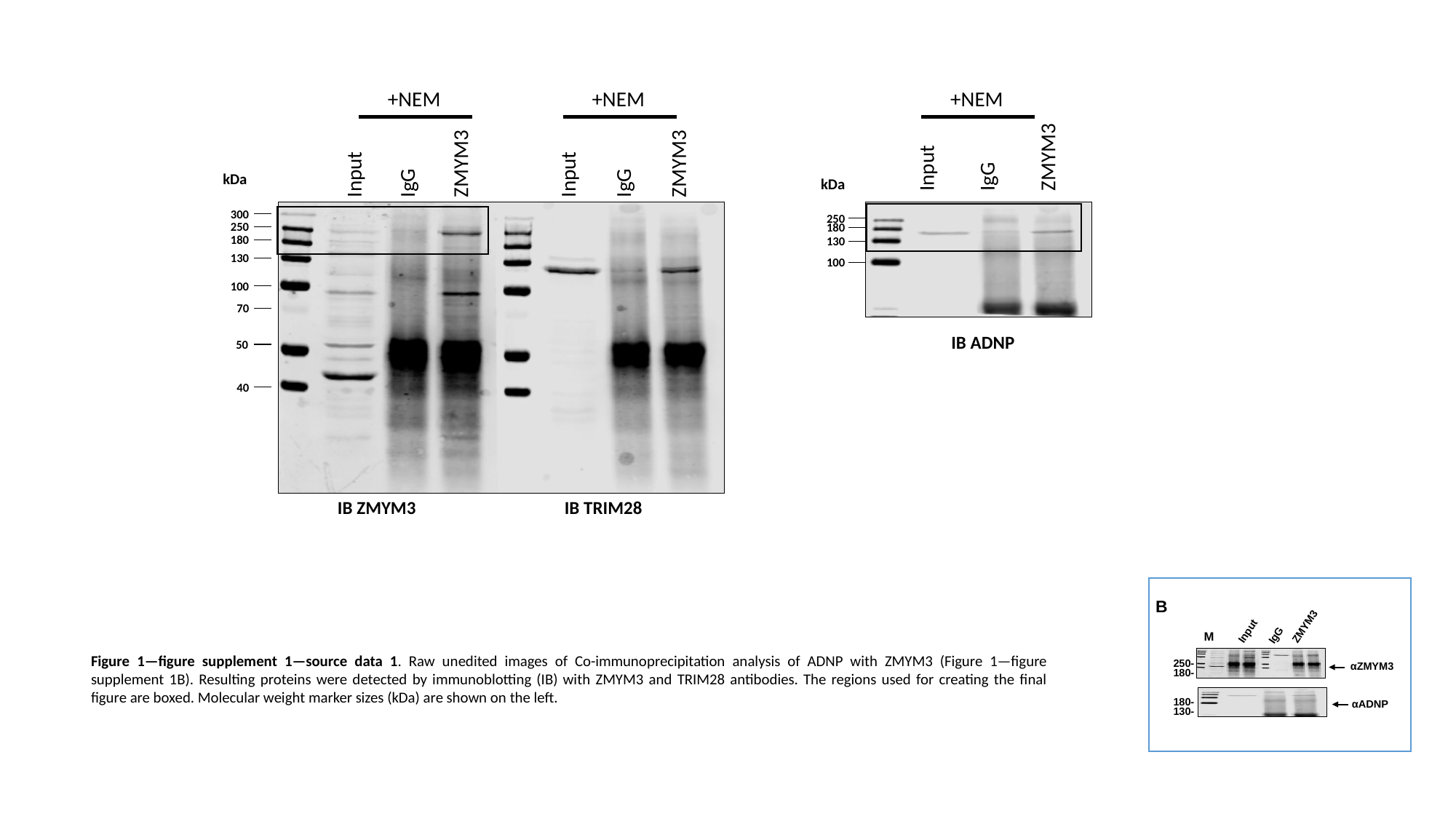

+NEM
+NEM
+NEM
 ZMYM3
 IgG
 Input
 ZMYM3
 ZMYM3
 IgG
 IgG
 Input
 Input
kDa
kDa
300
250
250
180
180
130
130
100
100
70
IB ADNP
50
40
IB ZMYM3
IB TRIM28
B
 ZMYM3
 Input
 IgG
M
Figure 1—figure supplement 1—source data 1. Raw unedited images of Co-immunoprecipitation analysis of ADNP with ZMYM3 (Figure 1—figure supplement 1B). Resulting proteins were detected by immunoblotting (IB) with ZMYM3 and TRIM28 antibodies. The regions used for creating the final figure are boxed. Molecular weight marker sizes (kDa) are shown on the left.
250-
αZMYM3
180-
180-
αADNP
130-
