# Supplementary material for: ZMYM2 controls human transposable element transcription through distinct co-regulatory complexes
Source: eLife. 2023 Nov 7;12:RP86669. doi: 10.7554/eLife.86669 (PMC10629813; doi:10.7554/eLife.86669)
Supplement: Figure 1—figure supplement 1—source data 2. — αTubulin was used as a loading control (bottom). Molecular weight markers (M) are shown. The regions used for creating the final figure are boxed. Molecular weight marker sizes (kDa) are shown on the right. [file elife-86669-fig1-figsupp1-data2.zip › Figure1S1Sourcedata2/Figure1S1Sourcedata2.pptx]

## Slide 1
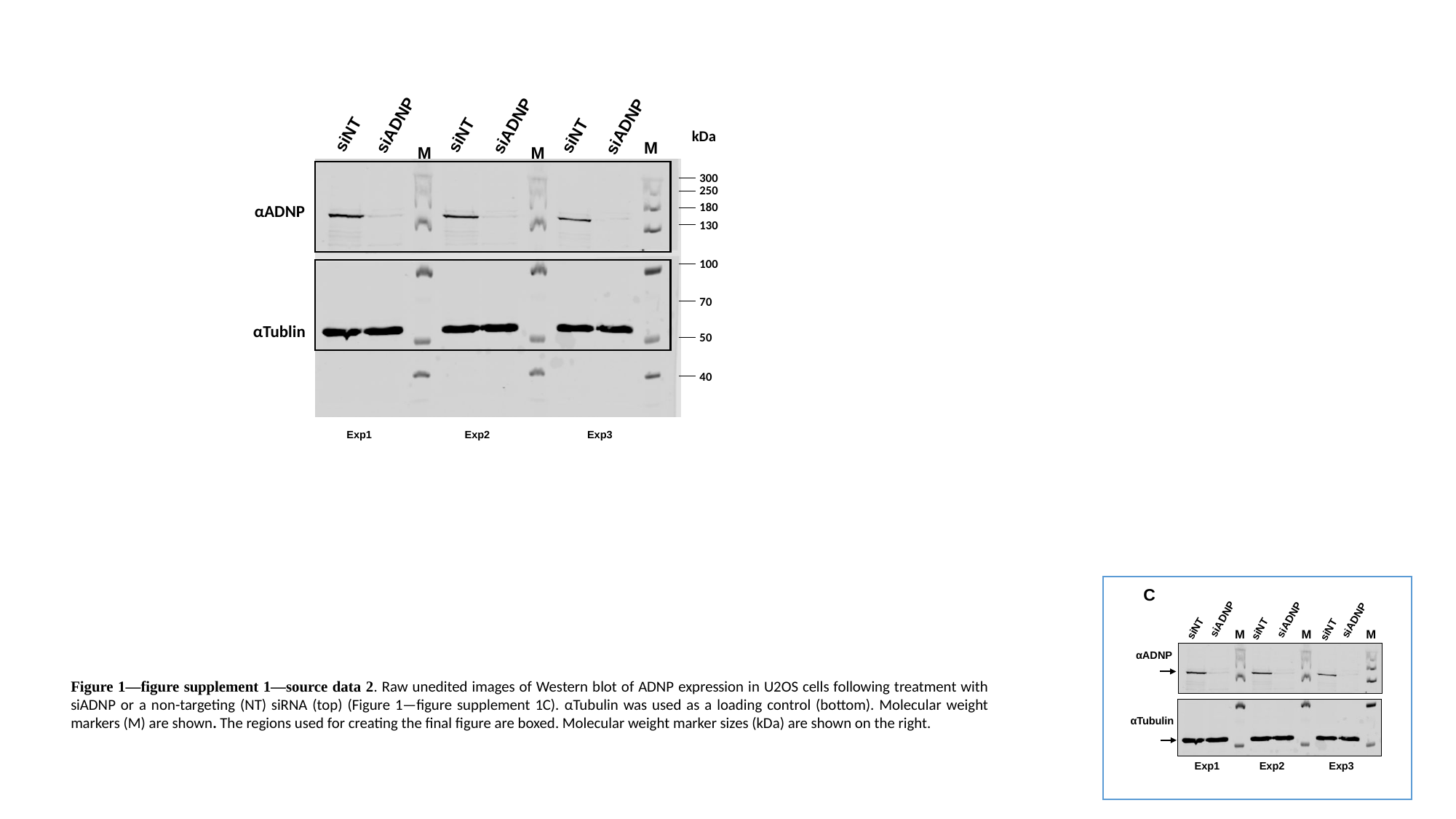

siADNP
siADNP
siADNP
 siNT
 siNT
 siNT
kDa
M
M
M
300
250
180
αADNP
130
100
70
αTublin
50
40
Exp1
Exp2
Exp3
C
siADNP
siADNP
siADNP
 siNT
 siNT
 siNT
M
M
M
αADNP
Figure 1—figure supplement 1—source data 2. Raw unedited images of Western blot of ADNP expression in U2OS cells following treatment with siADNP or a non-targeting (NT) siRNA (top) (Figure 1—figure supplement 1C). αTubulin was used as a loading control (bottom). Molecular weight markers (M) are shown. The regions used for creating the final figure are boxed. Molecular weight marker sizes (kDa) are shown on the right.
αTubulin
Exp1
Exp2
Exp3
