# Supplementary material for: ZMYM2 controls human transposable element transcription through distinct co-regulatory complexes
Source: eLife. 2023 Nov 7;12:RP86669. doi: 10.7554/eLife.86669 (PMC10629813; doi:10.7554/eLife.86669)
Supplement: Figure 3—figure supplement 1—source data 1. — Resulting proteins were detected by immunoblotting (IB) with ZMYM2 antibody. This is a longer exposure of Figure 4A top panel to better visualize the input protein. The regions used for creating the final figure are boxed. Molecular weight marker sizes (kDa) are shown on the left. [file elife-86669-fig3-figsupp1-data1.zip › Figure3S1Sourcedata1/Figure3S1Sourcedata1.pptx]

## Slide 1
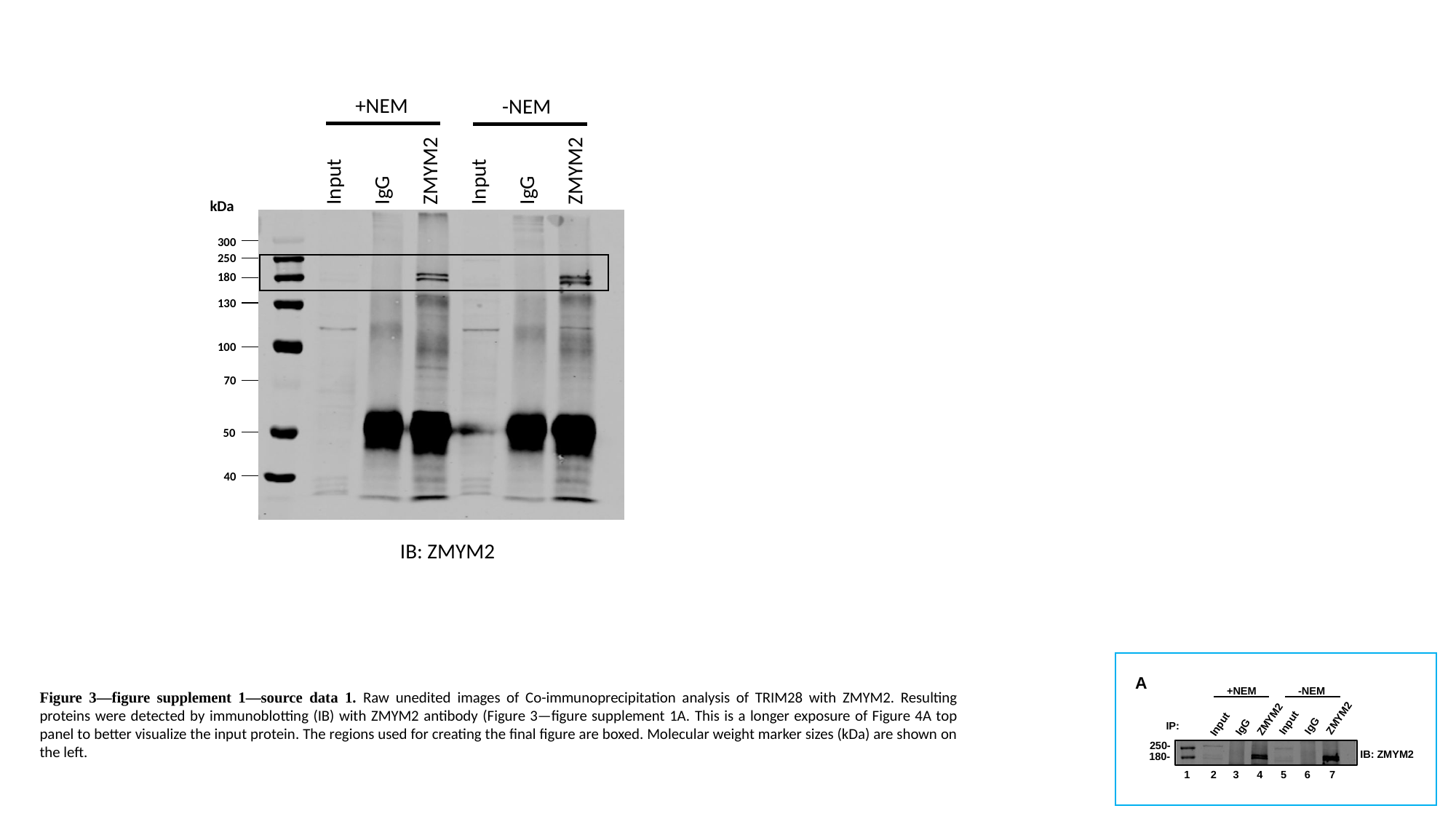

+NEM
-NEM
 Input
 ZMYM2
 ZMYM2
 IgG
 Input
 IgG
kDa
300
250
180
130
100
70
50
40
IB: ZMYM2
A
+NEM
-NEM
Figure 3—figure supplement 1—source data 1. Raw unedited images of Co-immunoprecipitation analysis of TRIM28 with ZMYM2. Resulting proteins were detected by immunoblotting (IB) with ZMYM2 antibody (Figure 3—figure supplement 1A. This is a longer exposure of Figure 4A top panel to better visualize the input protein. The regions used for creating the final figure are boxed. Molecular weight marker sizes (kDa) are shown on the left.
 ZMYM2
 ZMYM2
 Input
 Input
 IgG
IP:
 IgG
250-
IB: ZMYM2
180-
1
2
3
4
5
6
7
