# Supplementary material for: ZMYM2 controls human transposable element transcription through distinct co-regulatory complexes
Source: eLife. 2023 Nov 7;12:RP86669. doi: 10.7554/eLife.86669 (PMC10629813; doi:10.7554/eLife.86669)
Supplement: Figure 3—figure supplement 1—source data 2. — Resulting proteins were detected by immunoblotting (IB) with SUMO2 or TRIM28 antibodies. The regions used for creating the final figure are boxed. Molecular weight marker sizes (kDa) are shown on the left. [file elife-86669-fig3-figsupp1-data2.zip › Figure3S1Sourcedata2/Figure3S1Sourcedata2.pptx]

## Slide 1
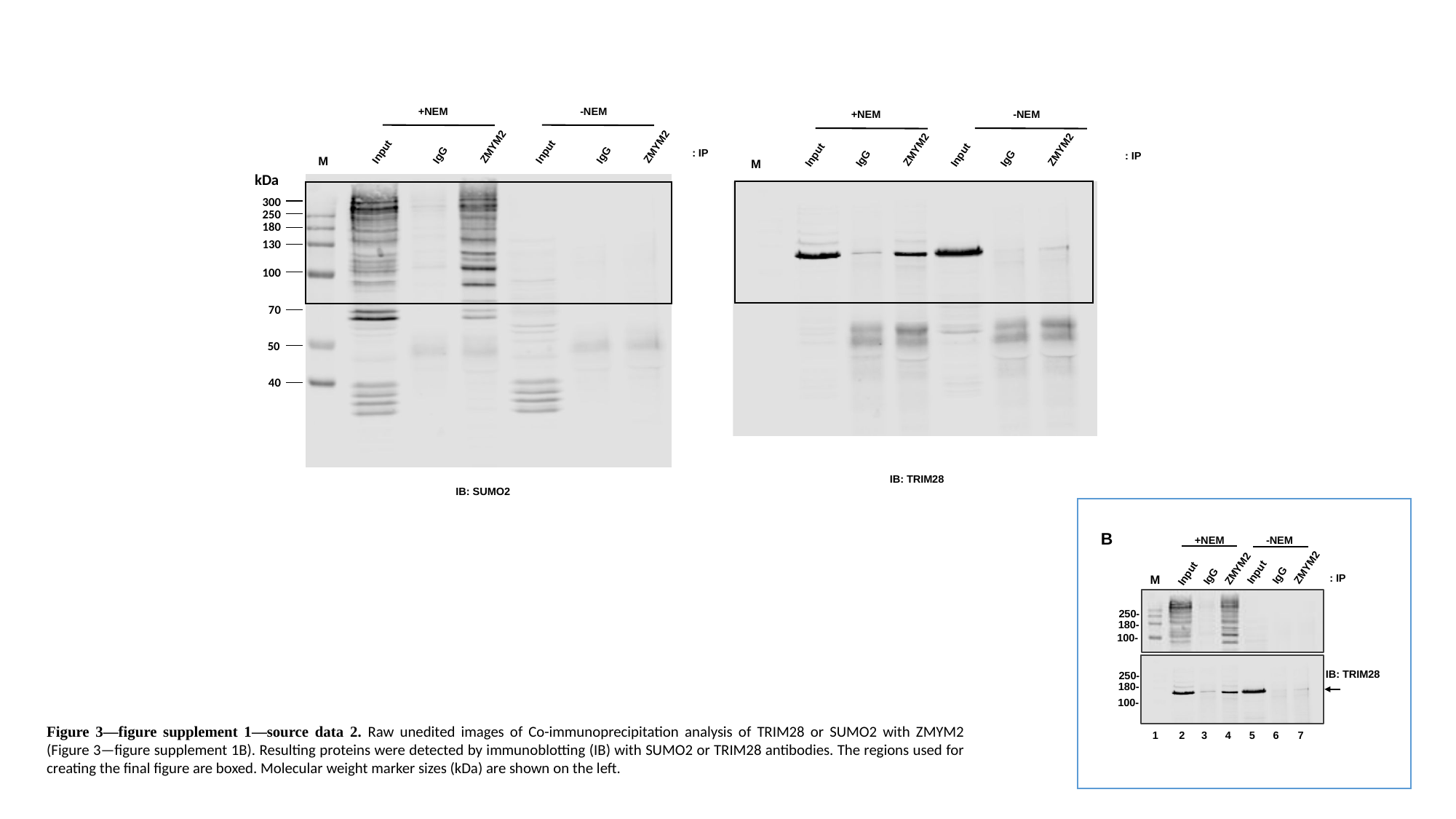

+NEM
-NEM
+NEM
-NEM
 ZMYM2
 ZMYM2
 Input
 Input
 ZMYM2
 ZMYM2
 Input
 Input
: IP
 IgG
 IgG
: IP
 IgG
 IgG
M
M
kDa
300
250
180
130
100
70
50
40
IB: TRIM28
IB: SUMO2
B
+NEM
-NEM
 ZMYM2
 ZMYM2
 Input
 Input
 IgG
 IgG
: IP
M
250-
180-
100-
IB: TRIM28
250-
180-
100-
Figure 3—figure supplement 1—source data 2. Raw unedited images of Co-immunoprecipitation analysis of TRIM28 or SUMO2 with ZMYM2 (Figure 3—figure supplement 1B). Resulting proteins were detected by immunoblotting (IB) with SUMO2 or TRIM28 antibodies. The regions used for creating the final figure are boxed. Molecular weight marker sizes (kDa) are shown on the left.
1
2
3
4
5
6
7
