# Supplementary material for: ZMYM2 controls human transposable element transcription through distinct co-regulatory complexes
Source: eLife. 2023 Nov 7;12:RP86669. doi: 10.7554/eLife.86669 (PMC10629813; doi:10.7554/eLife.86669)
Supplement: Figure 3—figure supplement 1—source data 3. — IPs (top) were immunoblotted (IB) with the indicated antibodies. Resulting proteins were detected by immunoblotting (IB) with GFP or TRIM28 antibodies. The regions used for creating the final figure are boxed. Molecular weight marker sizes (kDa) are shown on the left. [file elife-86669-fig3-figsupp1-data3.zip › Figure3S1Sourcedata3/Figure3S1Sourcedata3.pptx]

## Slide 1
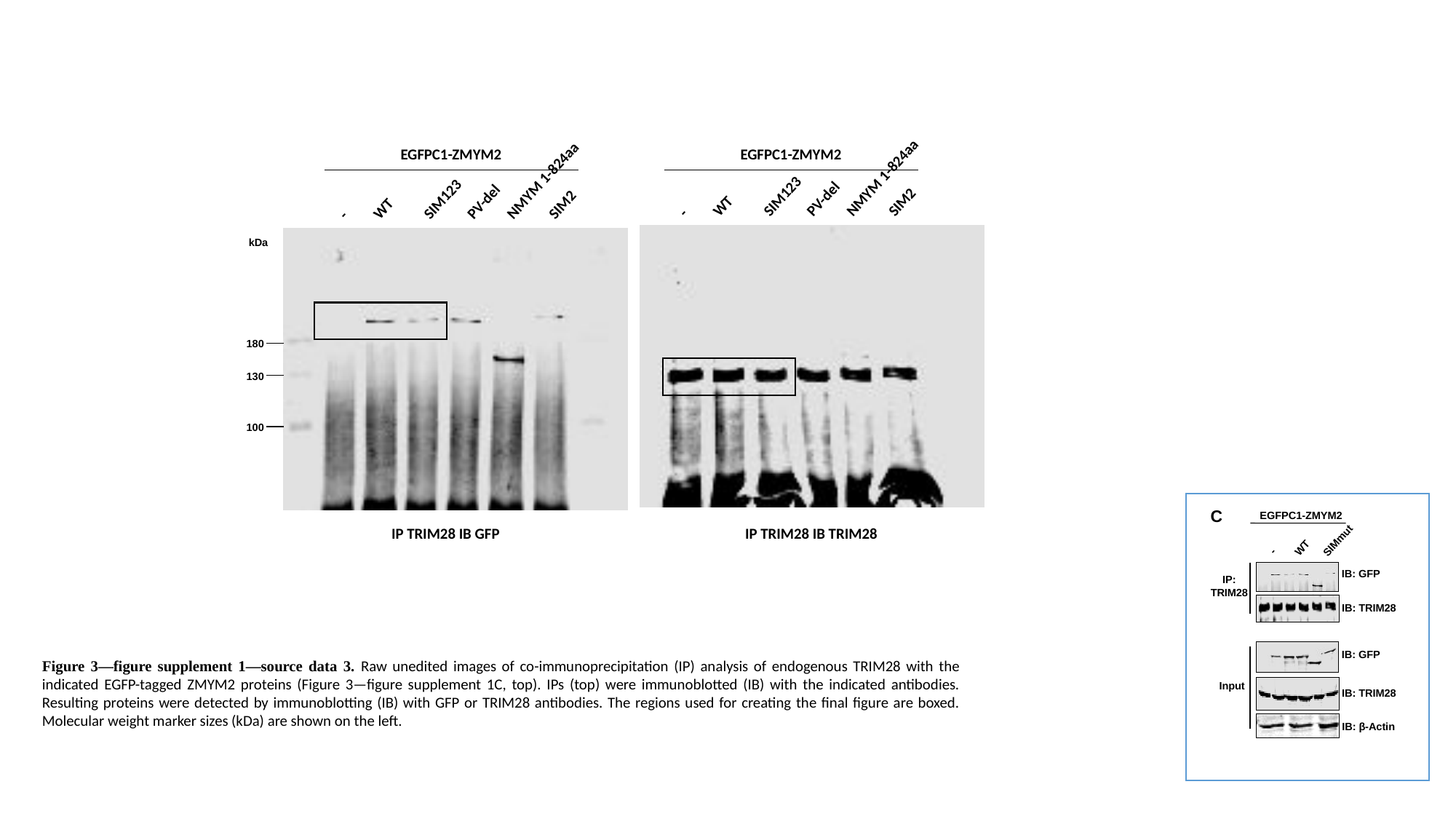

EGFPC1-ZMYM2
EGFPC1-ZMYM2
NMYM 1-824aa
NMYM 1-824aa
SIM123
PV-del
SIM123
PV-del
SIM2
SIM2
WT
WT
-
-
kDa
180
130
100
EGFPC1-ZMYM2
SIMmut
WT
-
IB: GFP
IP:
TRIM28
IB: TRIM28
IB: GFP
Input
IB: TRIM28
IB: β-Actin
C
IP TRIM28 IB GFP
IP TRIM28 IB TRIM28
Figure 3—figure supplement 1—source data 3. Raw unedited images of co-immunoprecipitation (IP) analysis of endogenous TRIM28 with the indicated EGFP-tagged ZMYM2 proteins (Figure 3—figure supplement 1C, top). IPs (top) were immunoblotted (IB) with the indicated antibodies. Resulting proteins were detected by immunoblotting (IB) with GFP or TRIM28 antibodies. The regions used for creating the final figure are boxed. Molecular weight marker sizes (kDa) are shown on the left.
