# Supplementary material for: ZMYM2 controls human transposable element transcription through distinct co-regulatory complexes
Source: eLife. 2023 Nov 7;12:RP86669. doi: 10.7554/eLife.86669 (PMC10629813; doi:10.7554/eLife.86669)
Supplement: Figure 3—figure supplement 1—source data 4. — Input samples were immunoblotted (IB) with the indicated antibodies. The regions used for creating the final figure are boxed. [file elife-86669-fig3-figsupp1-data4.zip › Figure3S1Sourcedata4/Figure3S1Sourcedata4.pptx]

## Slide 1
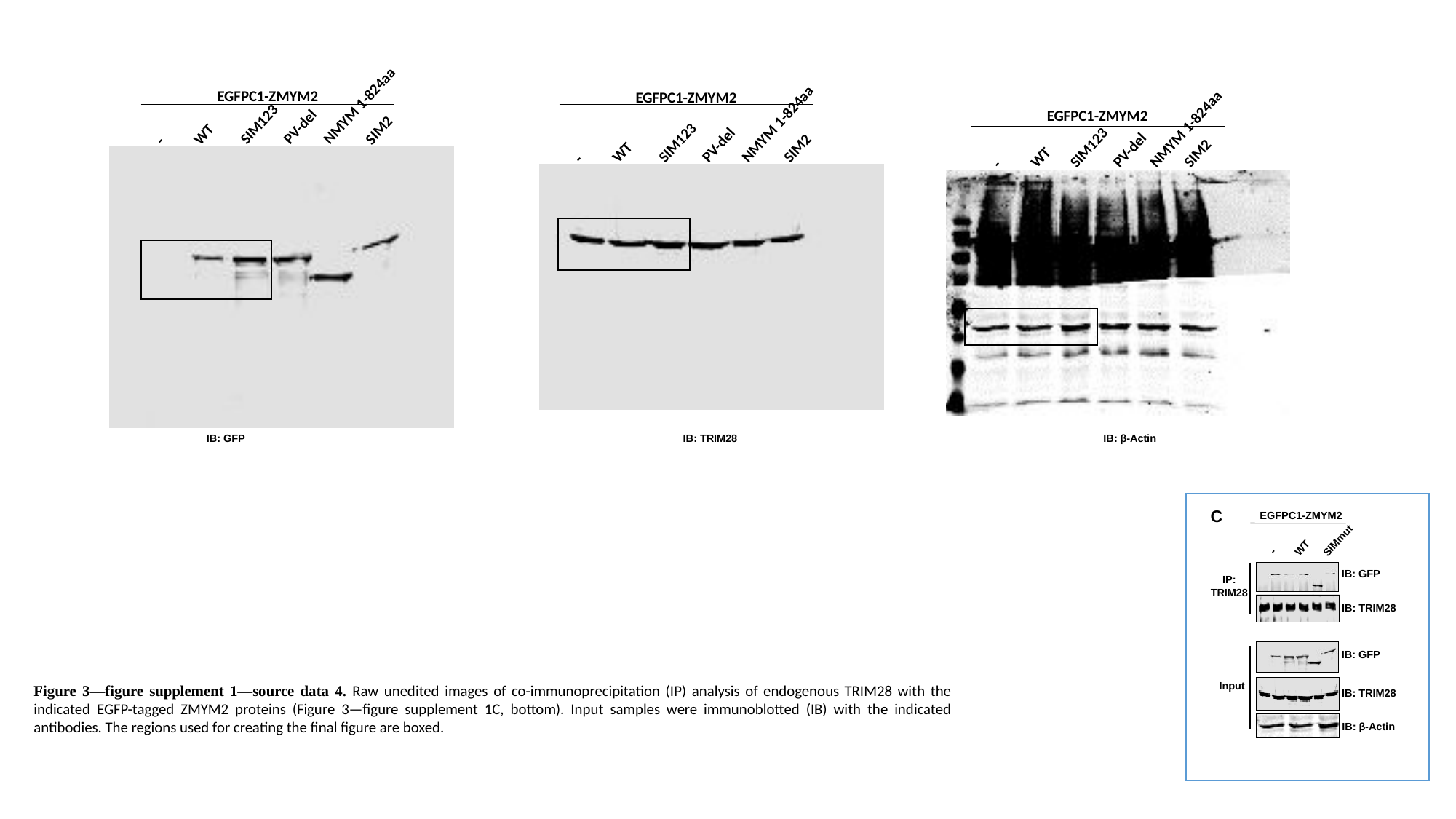

EGFPC1-ZMYM2
EGFPC1-ZMYM2
NMYM 1-824aa
EGFPC1-ZMYM2
NMYM 1-824aa
SIM123
PV-del
NMYM 1-824aa
SIM2
WT
-
SIM123
PV-del
SIM123
SIM2
PV-del
WT
SIM2
WT
-
-
IB: GFP
IB: TRIM28
IB: β-Actin
EGFPC1-ZMYM2
SIMmut
WT
-
IB: GFP
IP:
TRIM28
IB: TRIM28
IB: GFP
Input
IB: TRIM28
IB: β-Actin
C
Figure 3—figure supplement 1—source data 4. Raw unedited images of co-immunoprecipitation (IP) analysis of endogenous TRIM28 with the indicated EGFP-tagged ZMYM2 proteins (Figure 3—figure supplement 1C, bottom). Input samples were immunoblotted (IB) with the indicated antibodies. The regions used for creating the final figure are boxed.
