# Supplementary material for: ZMYM2 controls human transposable element transcription through distinct co-regulatory complexes
Source: eLife. 2023 Nov 7;12:RP86669. doi: 10.7554/eLife.86669 (PMC10629813; doi:10.7554/eLife.86669)
Supplement: Figure 3—figure supplement 1—source data 5. — Immunoprecipitation (IP) was performed with ZMYM2 or control IgG antibody from U2OS cells in the presence of NEM and the resulting TRIM28 detected by immunoblotting (IB). 10% input is shown. The regions used for creating the final figure are boxed. Molecular weight marker sizes (kDa) are shown on the left. [file elife-86669-fig3-figsupp1-data5.zip › Figure3S1Sourcedata5/Figure3S1Sourcedata5.pptx]

## Slide 1
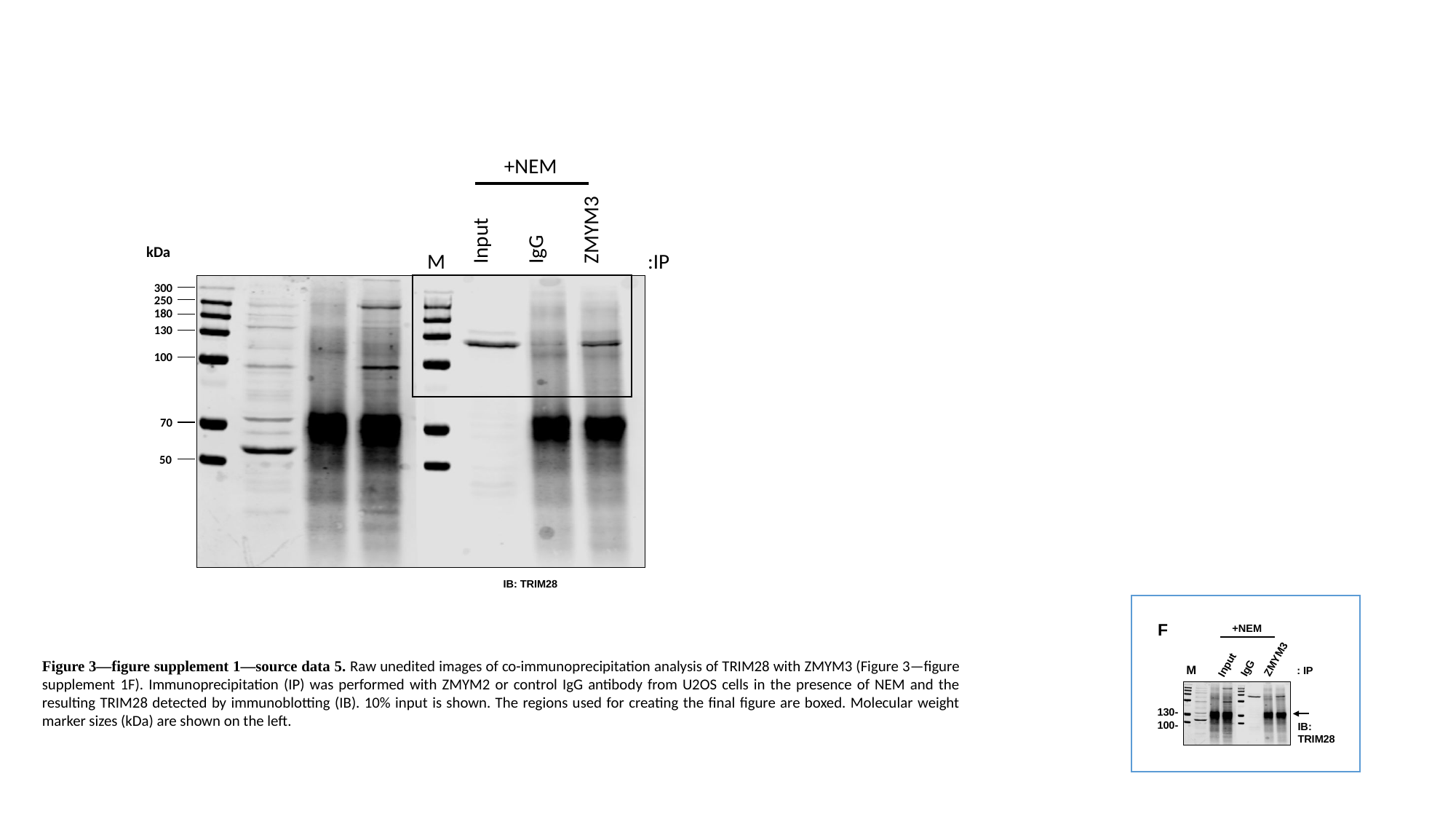

+NEM
 ZMYM3
 IgG
 Input
kDa
M
:IP
300
250
180
130
100
70
50
IB: TRIM28
+NEM
 ZMYM3
 IgG
 Input
IB: TRIM28
F
Figure 3—figure supplement 1—source data 5. Raw unedited images of co-immunoprecipitation analysis of TRIM28 with ZMYM3 (Figure 3—figure supplement 1F). Immunoprecipitation (IP) was performed with ZMYM2 or control IgG antibody from U2OS cells in the presence of NEM and the resulting TRIM28 detected by immunoblotting (IB). 10% input is shown. The regions used for creating the final figure are boxed. Molecular weight marker sizes (kDa) are shown on the left.
M
: IP
130-
100-
