# Supplementary material for: ZMYM2 controls human transposable element transcription through distinct co-regulatory complexes
Source: eLife. 2023 Nov 7;12:RP86669. doi: 10.7554/eLife.86669 (PMC10629813; doi:10.7554/eLife.86669)
Supplement: Figure 3—figure supplement 1—source data 6. — Lamin B (loading control), TRIM28 and ZMYM2 were detected by immunoblotting (IB) (Figure 3—figure supplement 1G). The regions used for creating the final figure are boxed. Molecular weight marker sizes (kDa) are shown on the left. [file elife-86669-fig3-figsupp1-data6.zip › Figure3S1Sourcedata6/Figure3S1Sourcedata6.pptx]

## Slide 1
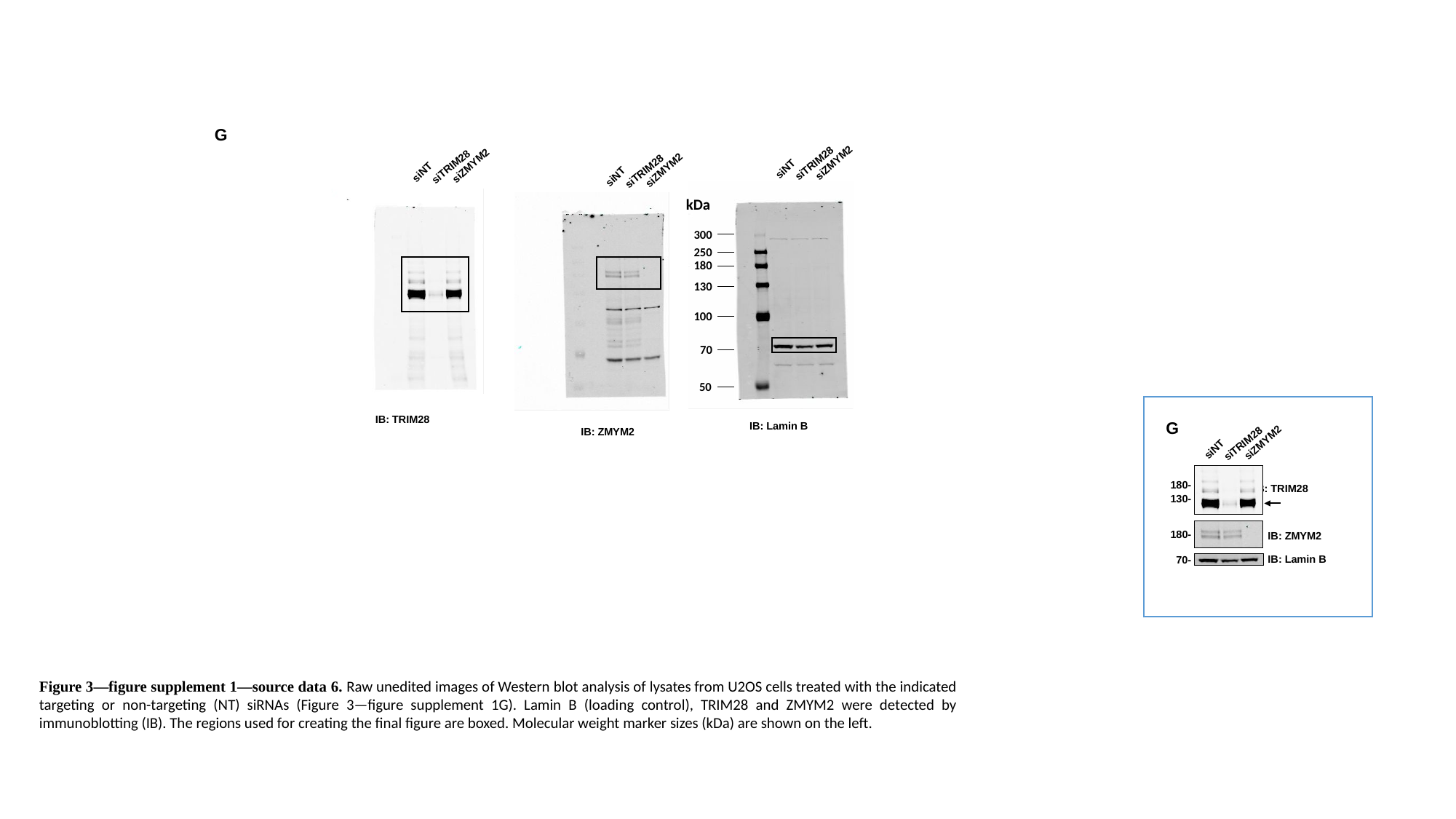

G
siZMYM2
siTRIM28
siZMYM2
siTRIM28
siZMYM2
siNT
siTRIM28
siNT
siNT
kDa
300
250
130-
180
130
100
70
50
IB: TRIM28
IB: Lamin B
G
siZMYM2
siTRIM28
siNT
180-
IB: TRIM28
130-
180-
IB: ZMYM2
IB: Lamin B
70-
IB: ZMYM2
Figure 3—figure supplement 1—source data 6. Raw unedited images of Western blot analysis of lysates from U2OS cells treated with the indicated targeting or non-targeting (NT) siRNAs (Figure 3—figure supplement 1G). Lamin B (loading control), TRIM28 and ZMYM2 were detected by immunoblotting (IB). The regions used for creating the final figure are boxed. Molecular weight marker sizes (kDa) are shown on the left.
