# Supplementary material for: ZMYM2 controls human transposable element transcription through distinct co-regulatory complexes
Source: eLife. 2023 Nov 7;12:RP86669. doi: 10.7554/eLife.86669 (PMC10629813; doi:10.7554/eLife.86669)
Supplement: Figure 4—source data 1. — Resulting proteins were detected by immunoblotting (IB) with ZMYM2 and TRIM28 antibodies. The regions used for creating the final figure are boxed. Molecular weight marker sizes (kDa) are shown on the left. [file elife-86669-fig4-data1.zip › Figure4Sourcedata1/Figure4Sourcedata1.pptx]

## Slide 1
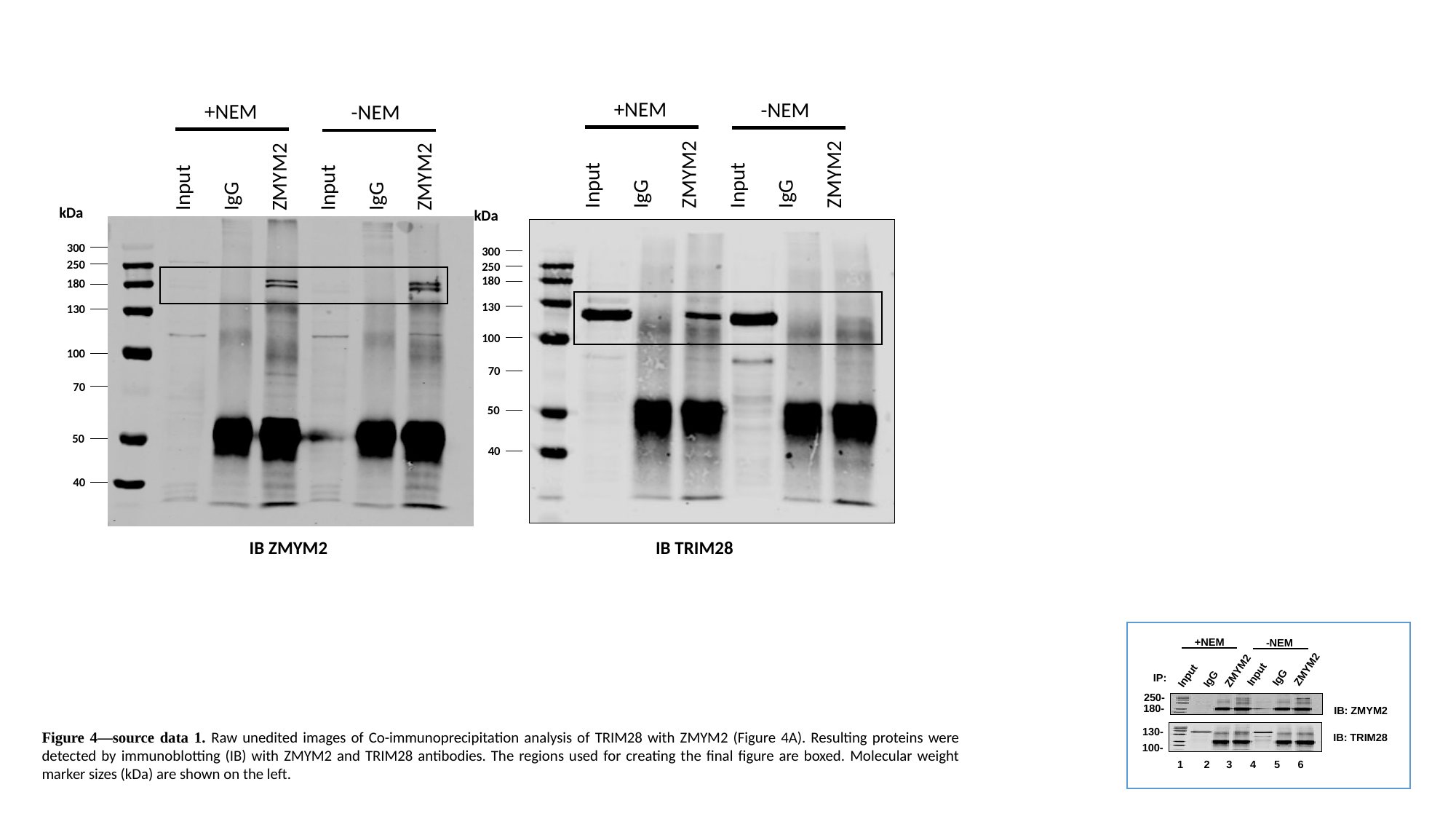

+NEM
-NEM
+NEM
-NEM
 Input
 Input
 ZMYM2
 ZMYM2
 ZMYM2
 ZMYM2
 IgG
 Input
 IgG
 Input
 IgG
 IgG
kDa
kDa
300
300
250
250
180
180
130
130
100
100
70
70
50
50
40
40
IB ZMYM2
IB TRIM28
+NEM
-NEM
 ZMYM2
 ZMYM2
 Input
 Input
 IgG
IP:
 IgG
250-
180-
IB: ZMYM2
130-
Figure 4—source data 1. Raw unedited images of Co-immunoprecipitation analysis of TRIM28 with ZMYM2 (Figure 4A). Resulting proteins were detected by immunoblotting (IB) with ZMYM2 and TRIM28 antibodies. The regions used for creating the final figure are boxed. Molecular weight marker sizes (kDa) are shown on the left.
IB: TRIM28
100-
1
2
3
4
5
6
