# Supplementary material for: ZMYM2 controls human transposable element transcription through distinct co-regulatory complexes
Source: eLife. 2023 Nov 7;12:RP86669. doi: 10.7554/eLife.86669 (PMC10629813; doi:10.7554/eLife.86669)
Supplement: Figure 5—figure supplement 1—source data 1. — β-actin is shown as a loading control (Figure 5—figure supplement 1A). The regions used for creating the final figure are boxed. Molecular weight marker sizes (kDa) are shown on the left. [file elife-86669-fig5-figsupp1-data1.zip › Figure5S1Sourcedata1/Figure5S1Sourcedata1.pptx]

## Slide 1
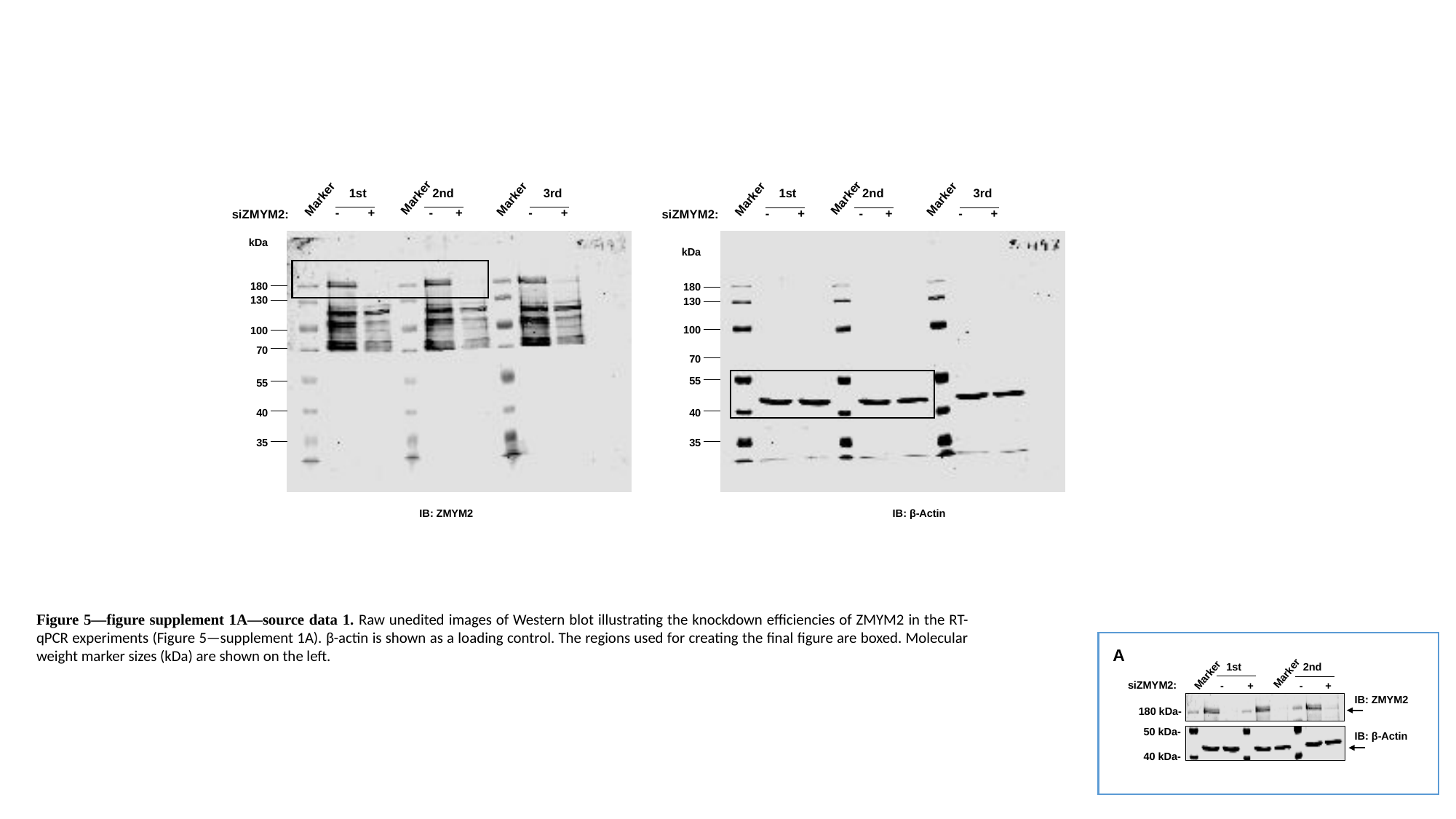

1st
2nd
3rd
1st
2nd
3rd
Marker
Marker
Marker
Marker
Marker
Marker
-
+
-
+
-
+
-
+
-
+
-
+
siZMYM2:
siZMYM2:
kDa
kDa
180
180
130
130
100
100
70
70
55
55
40
40
35
35
IB: ZMYM2
IB: β-Actin
Figure 5—figure supplement 1A—source data 1. Raw unedited images of Western blot illustrating the knockdown efficiencies of ZMYM2 in the RT-qPCR experiments (Figure 5—supplement 1A). β-actin is shown as a loading control. The regions used for creating the final figure are boxed. Molecular weight marker sizes (kDa) are shown on the left.
A
1st
2nd
siZMYM2:
-
+
-
+
IB: ZMYM2
IB: β-Actin
Marker
Marker
180 kDa-
50 kDa-
40 kDa-
