# Supplementary material for: ZMYM2 controls human transposable element transcription through distinct co-regulatory complexes
Source: eLife. 2023 Nov 7;12:RP86669. doi: 10.7554/eLife.86669 (PMC10629813; doi:10.7554/eLife.86669)
Supplement: Source data 1. [file elife-86669-data1.pptx]

## Slide 1
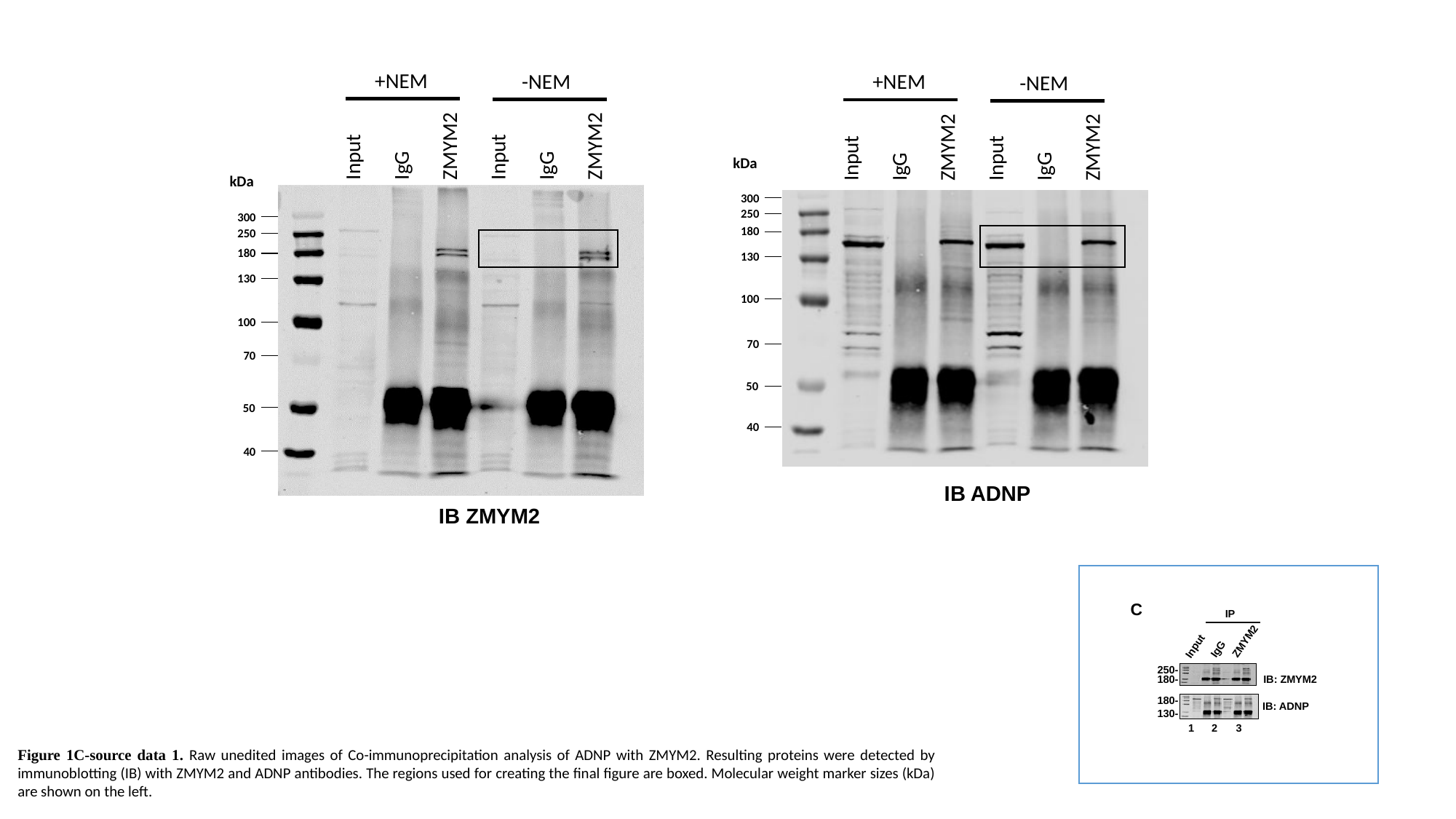

+NEM
-NEM
+NEM
-NEM
 Input
 Input
 ZMYM2
 ZMYM2
 ZMYM2
 ZMYM2
 IgG
 Input
 IgG
 Input
 IgG
 IgG
kDa
kDa
300
250
300
180
250
180
130
130
100
100
70
70
50
50
40
40
IB ADNP
IB ZMYM2
C
IP
 ZMYM2
 Input
 IgG
250-
180-
IB: ZMYM2
180-
IB: ADNP
130-
1
2
3
Figure 1C-source data 1. Raw unedited images of Co-immunoprecipitation analysis of ADNP with ZMYM2. Resulting proteins were detected by immunoblotting (IB) with ZMYM2 and ADNP antibodies. The regions used for creating the final figure are boxed. Molecular weight marker sizes (kDa) are shown on the left.

## Slide 2
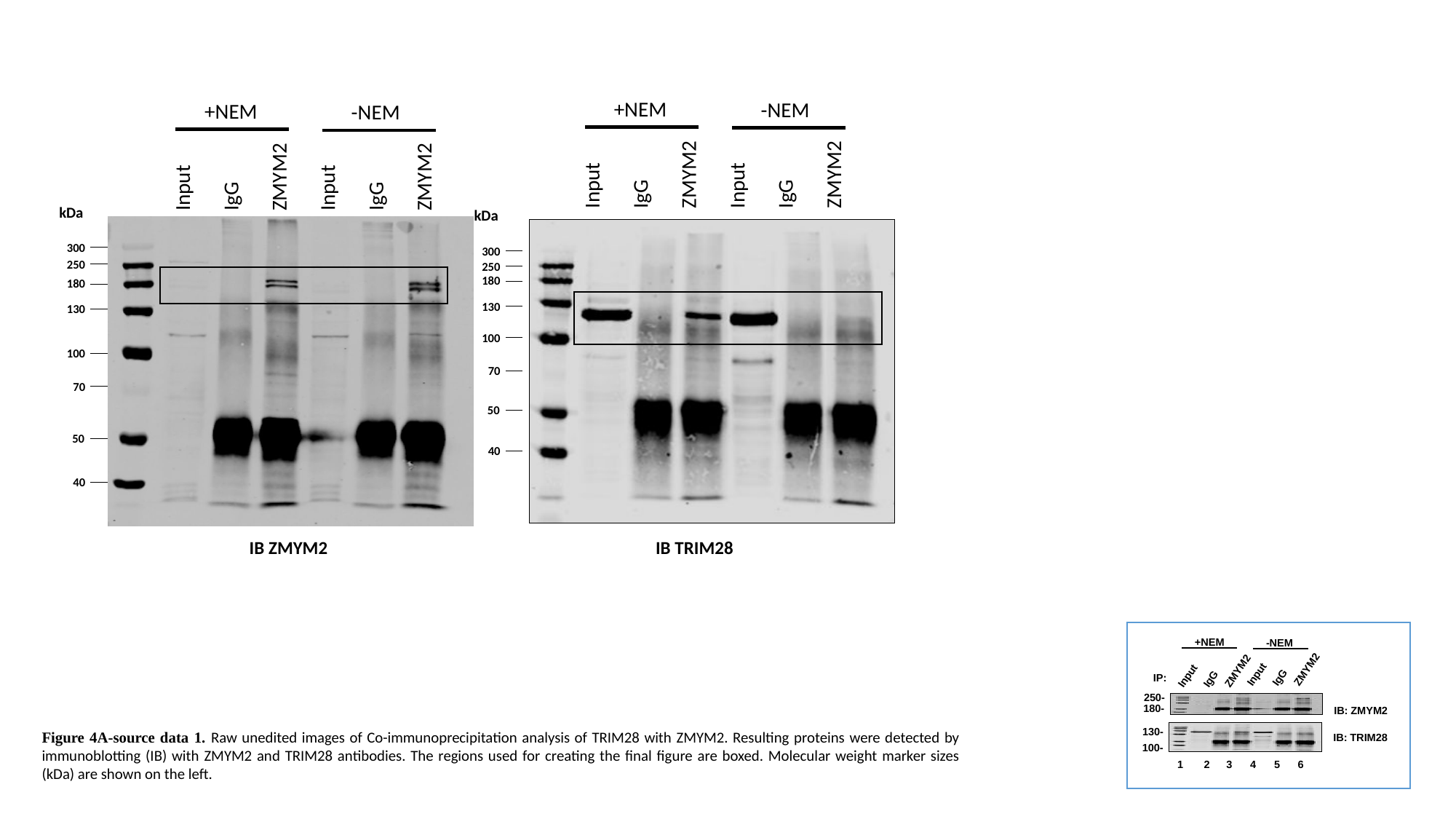

+NEM
-NEM
+NEM
-NEM
 Input
 Input
 ZMYM2
 ZMYM2
 ZMYM2
 ZMYM2
 IgG
 Input
 IgG
 Input
 IgG
 IgG
kDa
kDa
300
300
250
250
180
180
130
130
100
100
70
70
50
50
40
40
IB ZMYM2
IB TRIM28
+NEM
-NEM
 ZMYM2
 ZMYM2
 Input
 Input
 IgG
IP:
 IgG
250-
180-
IB: ZMYM2
130-
Figure 4A-source data 1. Raw unedited images of Co-immunoprecipitation analysis of TRIM28 with ZMYM2. Resulting proteins were detected by immunoblotting (IB) with ZMYM2 and TRIM28 antibodies. The regions used for creating the final figure are boxed. Molecular weight marker sizes (kDa) are shown on the left.
IB: TRIM28
100-
1
2
3
4
5
6

## Slide 3
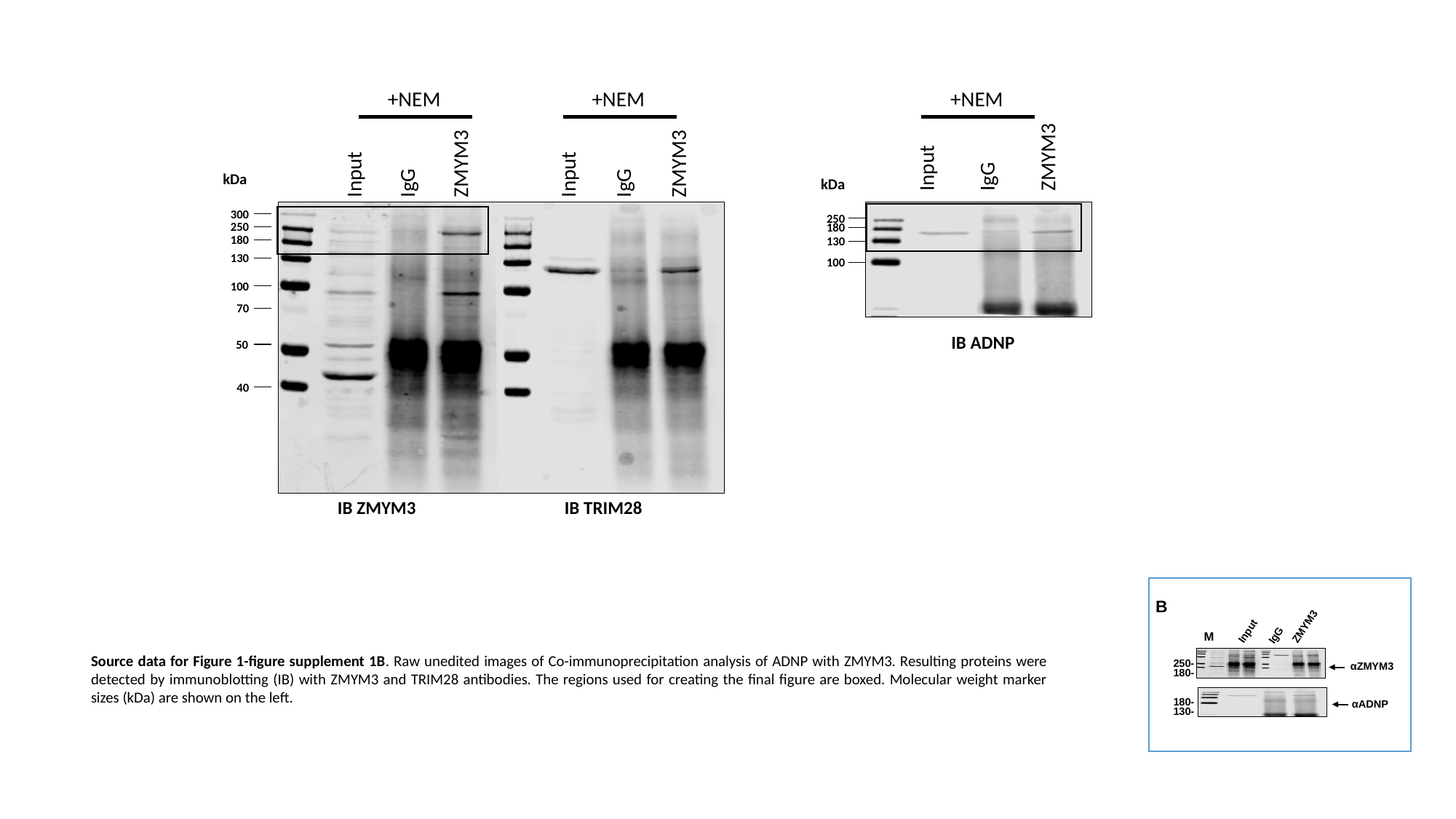

+NEM
+NEM
+NEM
 ZMYM3
 IgG
 Input
 ZMYM3
 ZMYM3
 IgG
 IgG
 Input
 Input
kDa
kDa
300
250
250
180
180
130
130
100
100
70
IB ADNP
50
40
IB ZMYM3
IB TRIM28
B
 ZMYM3
 Input
 IgG
M
Source data for Figure 1-figure supplement 1B. Raw unedited images of Co-immunoprecipitation analysis of ADNP with ZMYM3. Resulting proteins were detected by immunoblotting (IB) with ZMYM3 and TRIM28 antibodies. The regions used for creating the final figure are boxed. Molecular weight marker sizes (kDa) are shown on the left.
250-
αZMYM3
180-
180-
αADNP
130-

## Slide 4
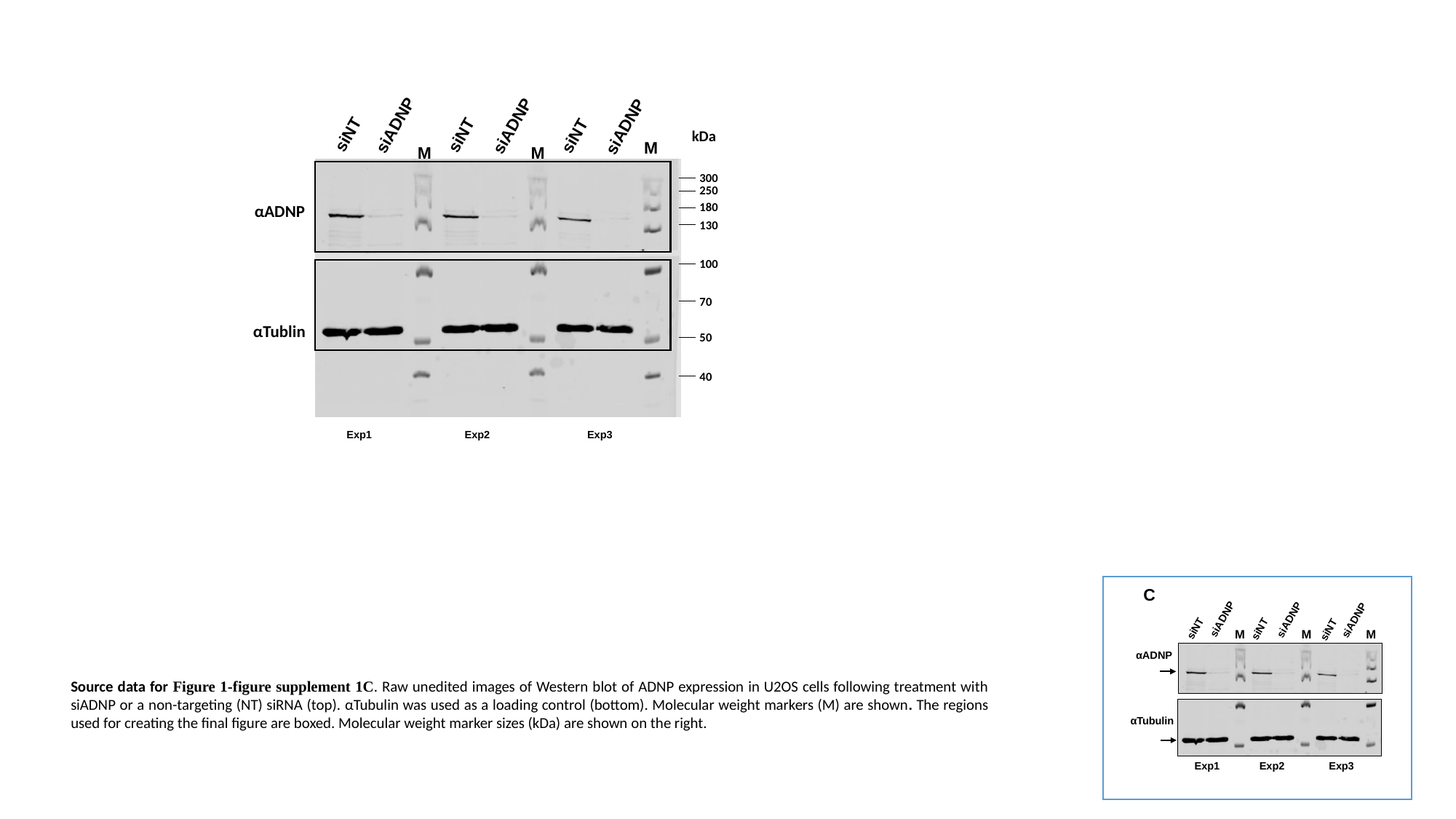

siADNP
siADNP
siADNP
 siNT
 siNT
 siNT
kDa
M
M
M
300
250
180
αADNP
130
100
70
αTublin
50
40
Exp1
Exp2
Exp3
C
siADNP
siADNP
siADNP
 siNT
 siNT
 siNT
M
M
M
αADNP
Source data for Figure 1-figure supplement 1C. Raw unedited images of Western blot of ADNP expression in U2OS cells following treatment with siADNP or a non-targeting (NT) siRNA (top). αTubulin was used as a loading control (bottom). Molecular weight markers (M) are shown. The regions used for creating the final figure are boxed. Molecular weight marker sizes (kDa) are shown on the right.
αTubulin
Exp1
Exp2
Exp3

## Slide 5
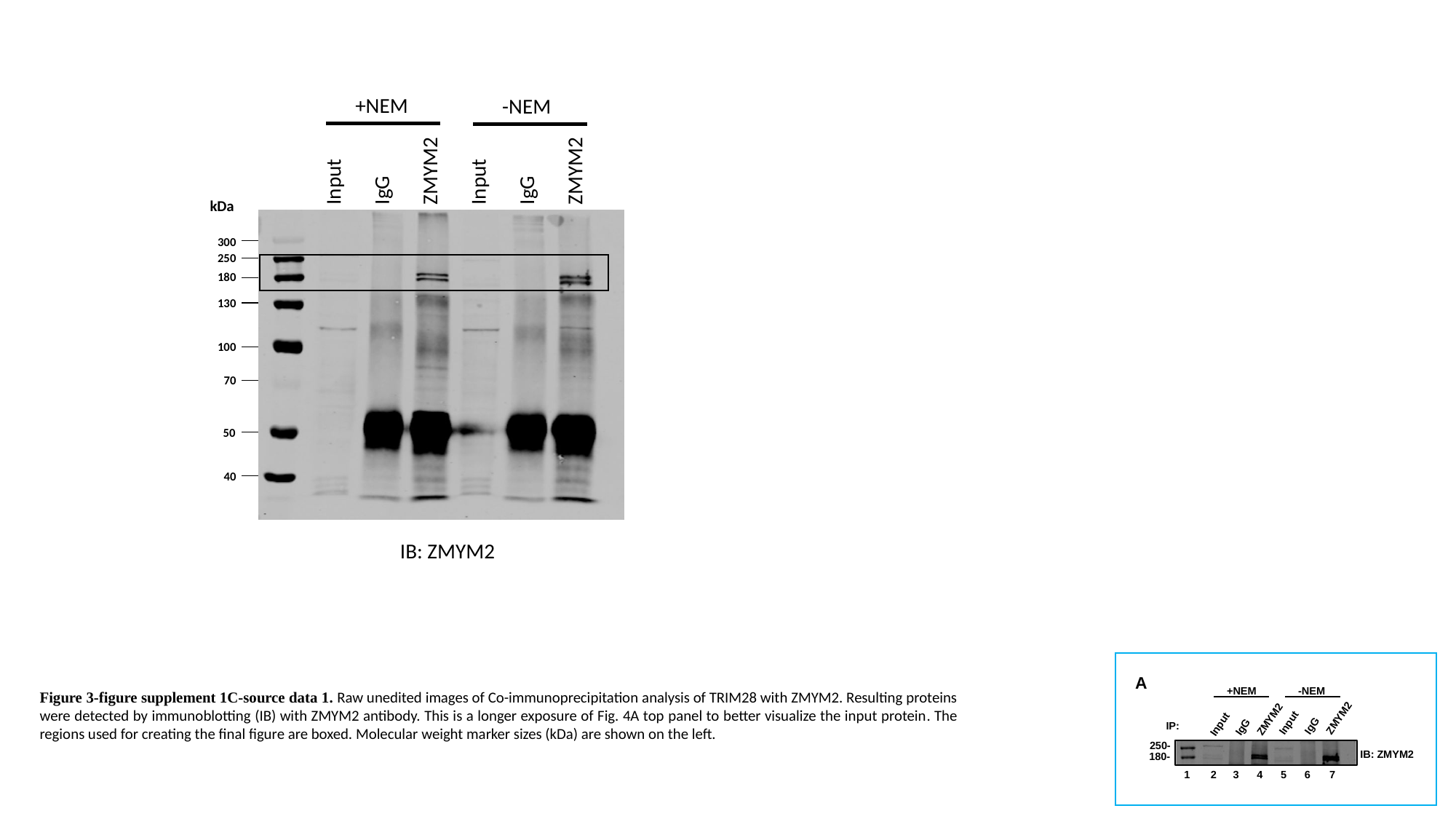

+NEM
-NEM
 Input
 ZMYM2
 ZMYM2
 IgG
 Input
 IgG
kDa
300
250
180
130
100
70
50
40
IB: ZMYM2
A
+NEM
-NEM
Figure 3-figure supplement 1C-source data 1. Raw unedited images of Co-immunoprecipitation analysis of TRIM28 with ZMYM2. Resulting proteins were detected by immunoblotting (IB) with ZMYM2 antibody. This is a longer exposure of Fig. 4A top panel to better visualize the input protein. The regions used for creating the final figure are boxed. Molecular weight marker sizes (kDa) are shown on the left.
 ZMYM2
 ZMYM2
 Input
 Input
 IgG
IP:
 IgG
250-
IB: ZMYM2
180-
1
2
3
4
5
6
7

## Slide 6
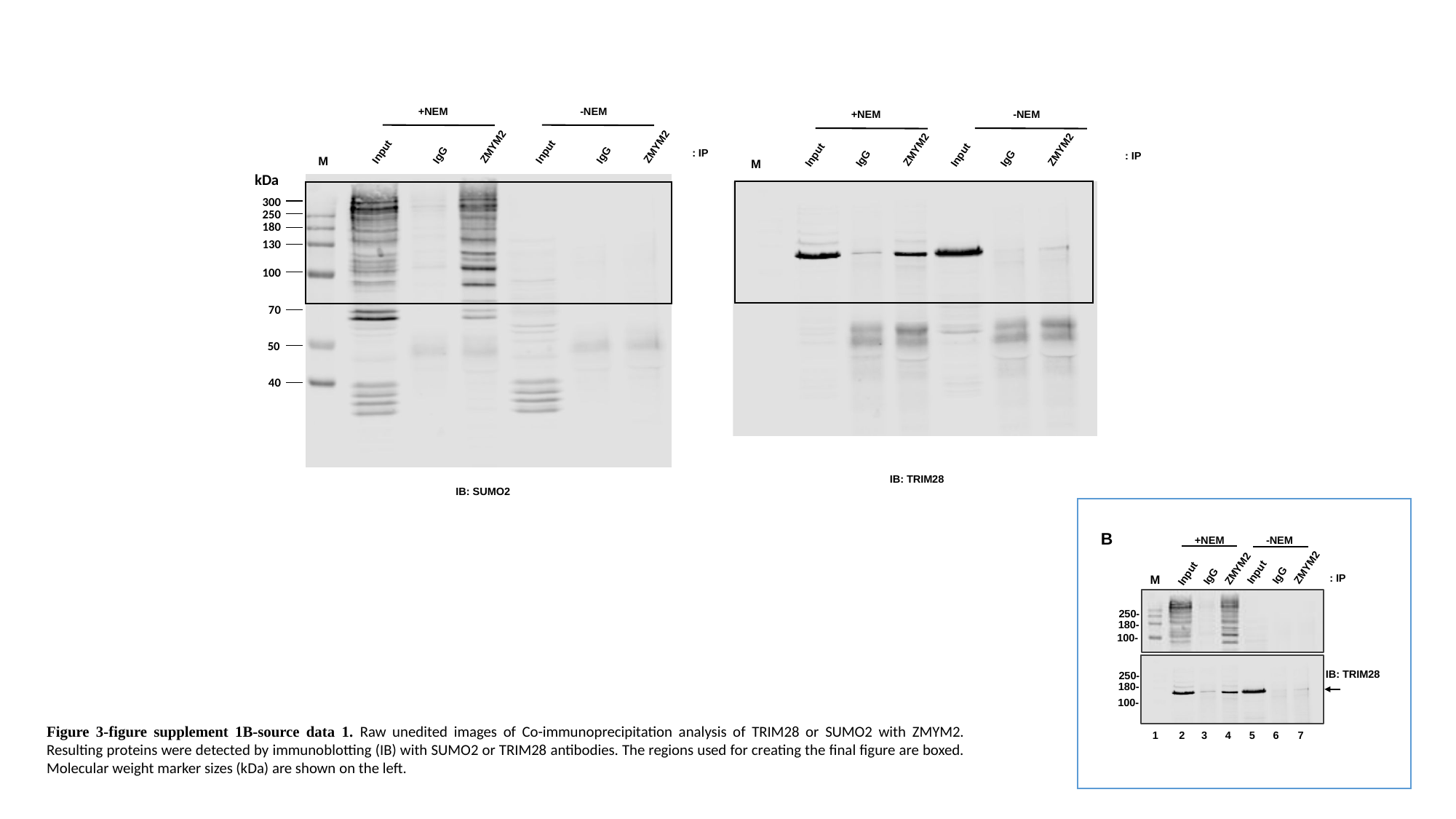

+NEM
-NEM
+NEM
-NEM
 ZMYM2
 ZMYM2
 Input
 Input
 ZMYM2
 ZMYM2
 Input
 Input
: IP
 IgG
 IgG
: IP
 IgG
 IgG
M
M
kDa
300
250
180
130
100
70
50
40
IB: TRIM28
IB: SUMO2
B
+NEM
-NEM
 ZMYM2
 ZMYM2
 Input
 Input
 IgG
 IgG
: IP
M
250-
180-
100-
IB: TRIM28
250-
180-
100-
Figure 3-figure supplement 1B-source data 1. Raw unedited images of Co-immunoprecipitation analysis of TRIM28 or SUMO2 with ZMYM2. Resulting proteins were detected by immunoblotting (IB) with SUMO2 or TRIM28 antibodies. The regions used for creating the final figure are boxed. Molecular weight marker sizes (kDa) are shown on the left.
1
2
3
4
5
6
7

## Slide 7
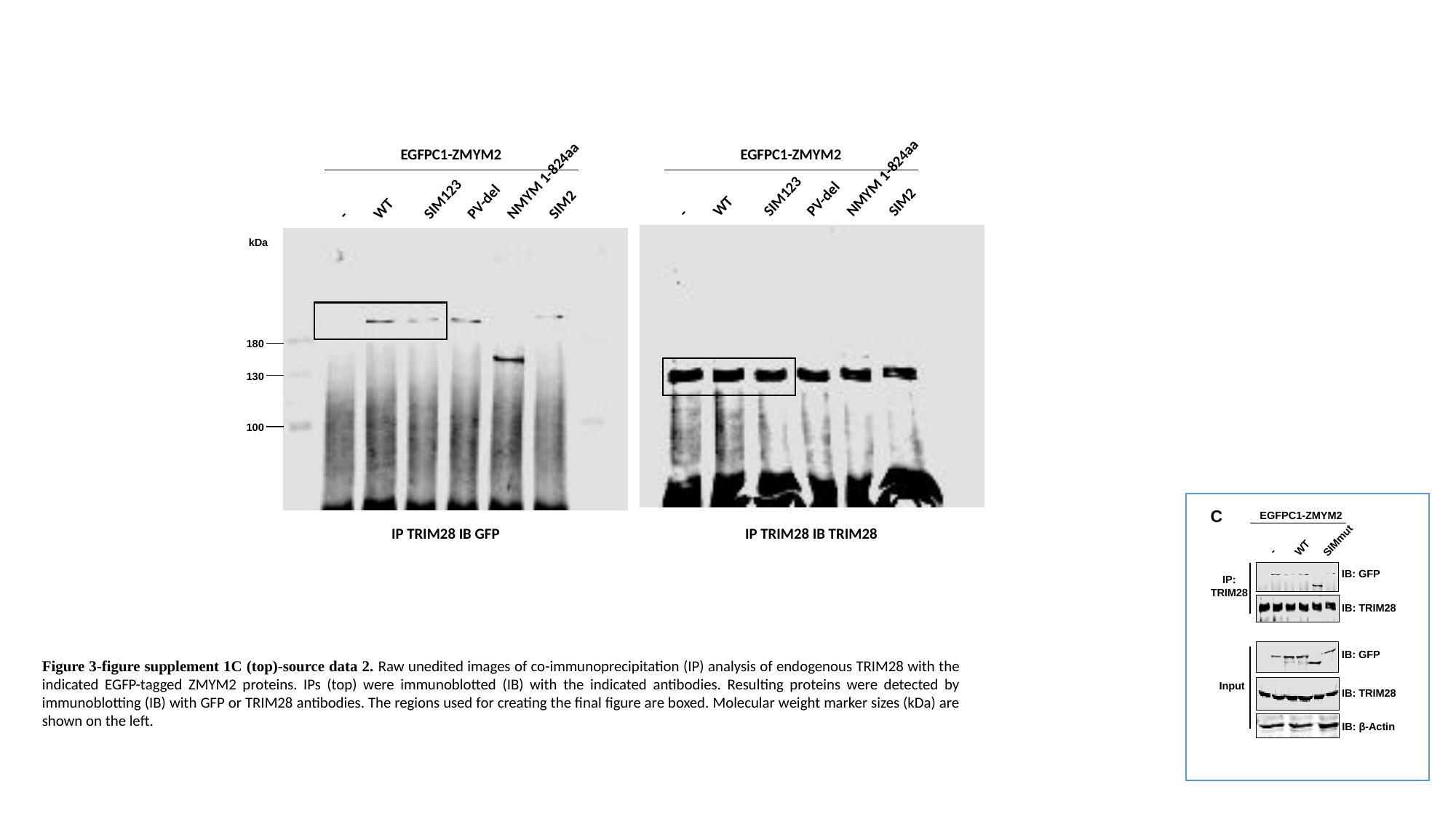

EGFPC1-ZMYM2
EGFPC1-ZMYM2
NMYM 1-824aa
NMYM 1-824aa
SIM123
PV-del
SIM123
PV-del
SIM2
SIM2
WT
WT
-
-
kDa
180
130
100
EGFPC1-ZMYM2
SIMmut
WT
-
IB: GFP
IP:
TRIM28
IB: TRIM28
IB: GFP
Input
IB: TRIM28
IB: β-Actin
C
IP TRIM28 IB GFP
IP TRIM28 IB TRIM28
Figure 3-figure supplement 1C (top)-source data 2. Raw unedited images of co-immunoprecipitation (IP) analysis of endogenous TRIM28 with the indicated EGFP-tagged ZMYM2 proteins. IPs (top) were immunoblotted (IB) with the indicated antibodies. Resulting proteins were detected by immunoblotting (IB) with GFP or TRIM28 antibodies. The regions used for creating the final figure are boxed. Molecular weight marker sizes (kDa) are shown on the left.

## Slide 8
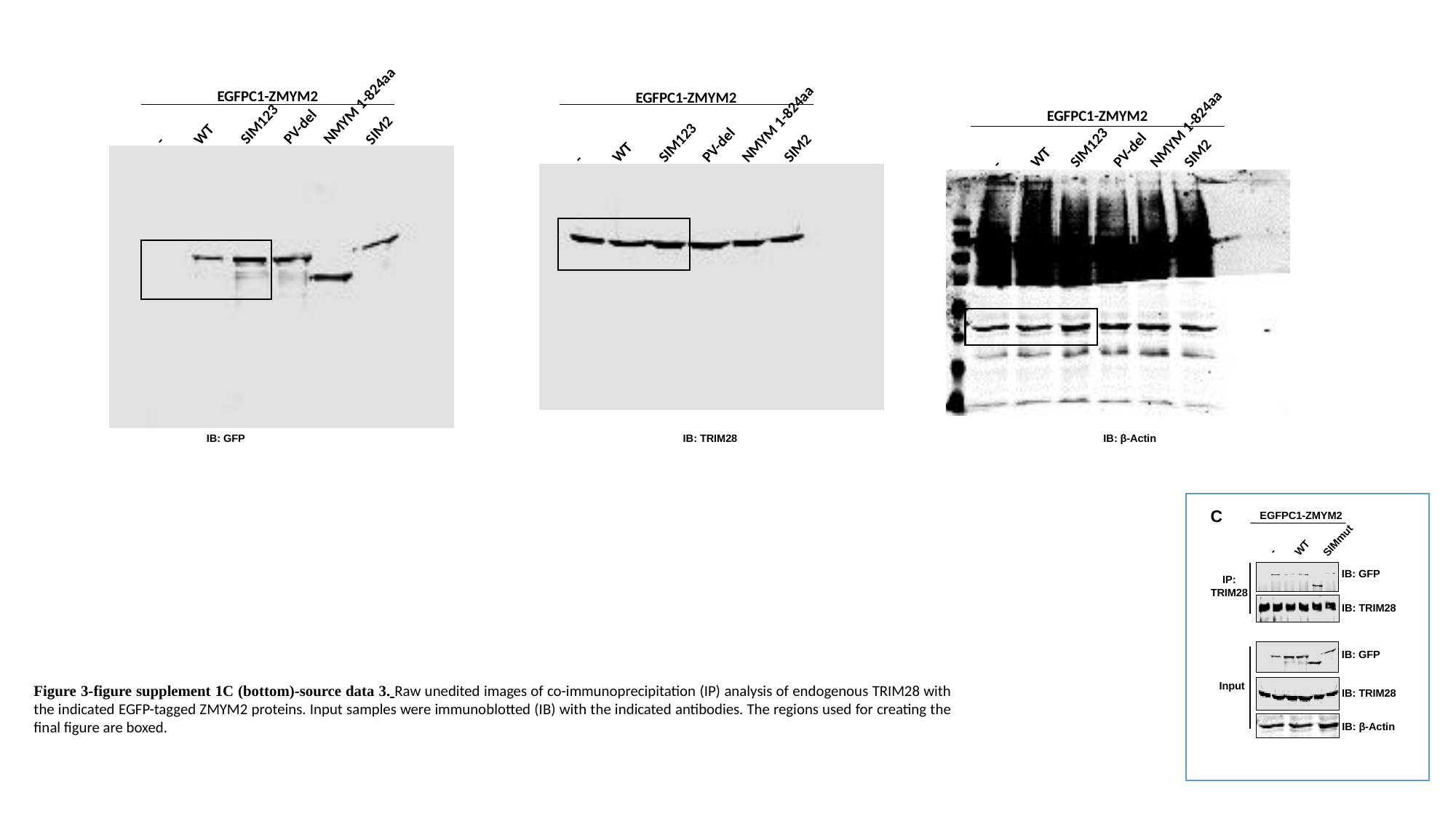

EGFPC1-ZMYM2
EGFPC1-ZMYM2
NMYM 1-824aa
EGFPC1-ZMYM2
NMYM 1-824aa
SIM123
PV-del
NMYM 1-824aa
SIM2
WT
-
SIM123
PV-del
SIM123
SIM2
PV-del
WT
SIM2
WT
-
-
IB: GFP
IB: TRIM28
IB: β-Actin
EGFPC1-ZMYM2
SIMmut
WT
-
IB: GFP
IP:
TRIM28
IB: TRIM28
IB: GFP
Input
IB: TRIM28
IB: β-Actin
C
Figure 3-figure supplement 1C (bottom)-source data 3. Raw unedited images of co-immunoprecipitation (IP) analysis of endogenous TRIM28 with the indicated EGFP-tagged ZMYM2 proteins. Input samples were immunoblotted (IB) with the indicated antibodies. The regions used for creating the final figure are boxed.

## Slide 9
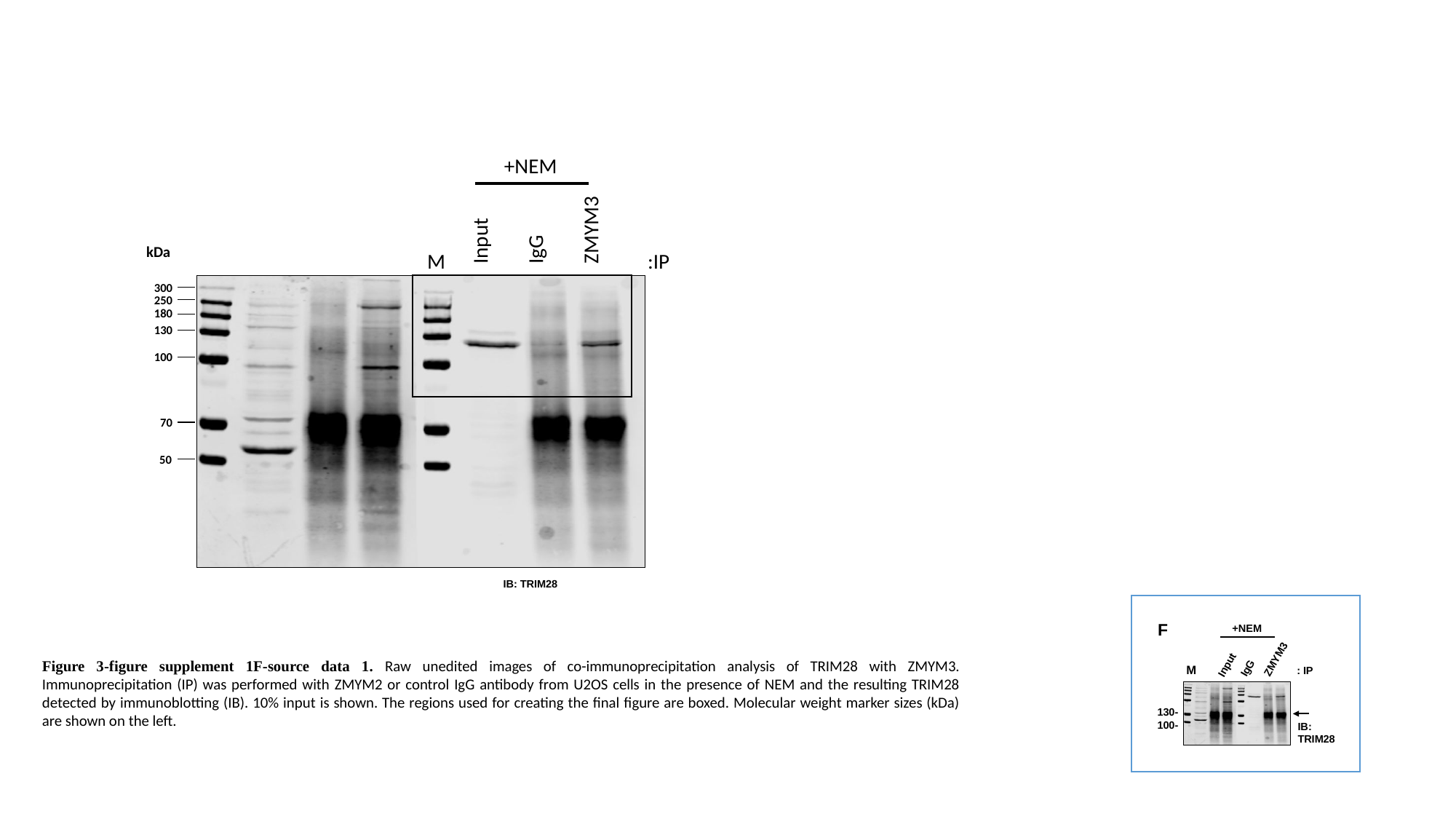

+NEM
 ZMYM3
 IgG
 Input
kDa
M
:IP
300
250
180
130
100
70
50
IB: TRIM28
+NEM
 ZMYM3
 IgG
 Input
IB: TRIM28
F
Figure 3-figure supplement 1F-source data 1. Raw unedited images of co-immunoprecipitation analysis of TRIM28 with ZMYM3. Immunoprecipitation (IP) was performed with ZMYM2 or control IgG antibody from U2OS cells in the presence of NEM and the resulting TRIM28 detected by immunoblotting (IB). 10% input is shown. The regions used for creating the final figure are boxed. Molecular weight marker sizes (kDa) are shown on the left.
M
: IP
130-
100-

## Slide 10
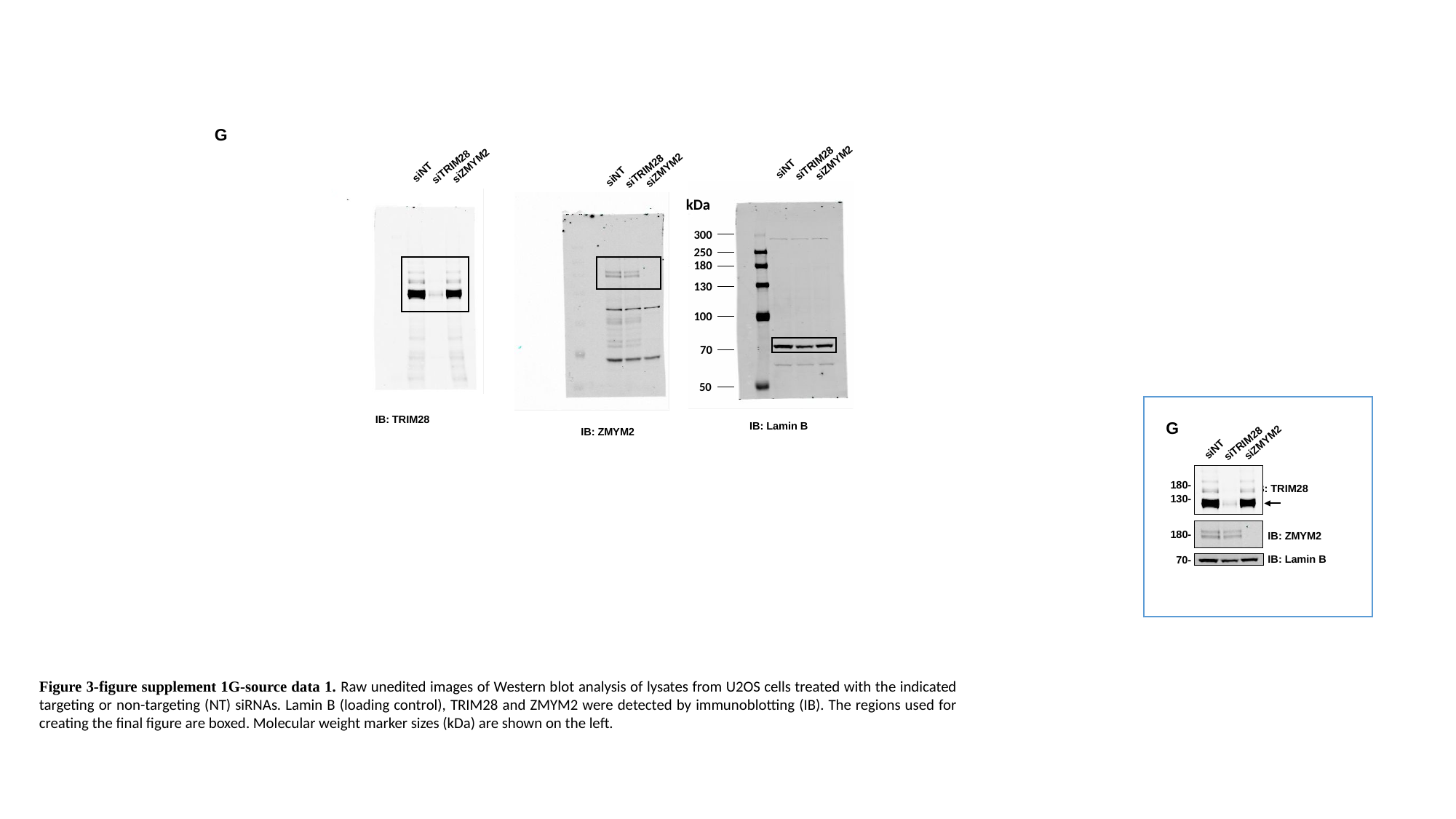

G
siZMYM2
siTRIM28
siZMYM2
siTRIM28
siZMYM2
siNT
siTRIM28
siNT
siNT
kDa
300
250
130-
180
130
100
70
50
IB: TRIM28
IB: Lamin B
G
siZMYM2
siTRIM28
siNT
180-
IB: TRIM28
130-
180-
IB: ZMYM2
IB: Lamin B
70-
IB: ZMYM2
Figure 3-figure supplement 1G-source data 1. Raw unedited images of Western blot analysis of lysates from U2OS cells treated with the indicated targeting or non-targeting (NT) siRNAs. Lamin B (loading control), TRIM28 and ZMYM2 were detected by immunoblotting (IB). The regions used for creating the final figure are boxed. Molecular weight marker sizes (kDa) are shown on the left.

## Slide 11
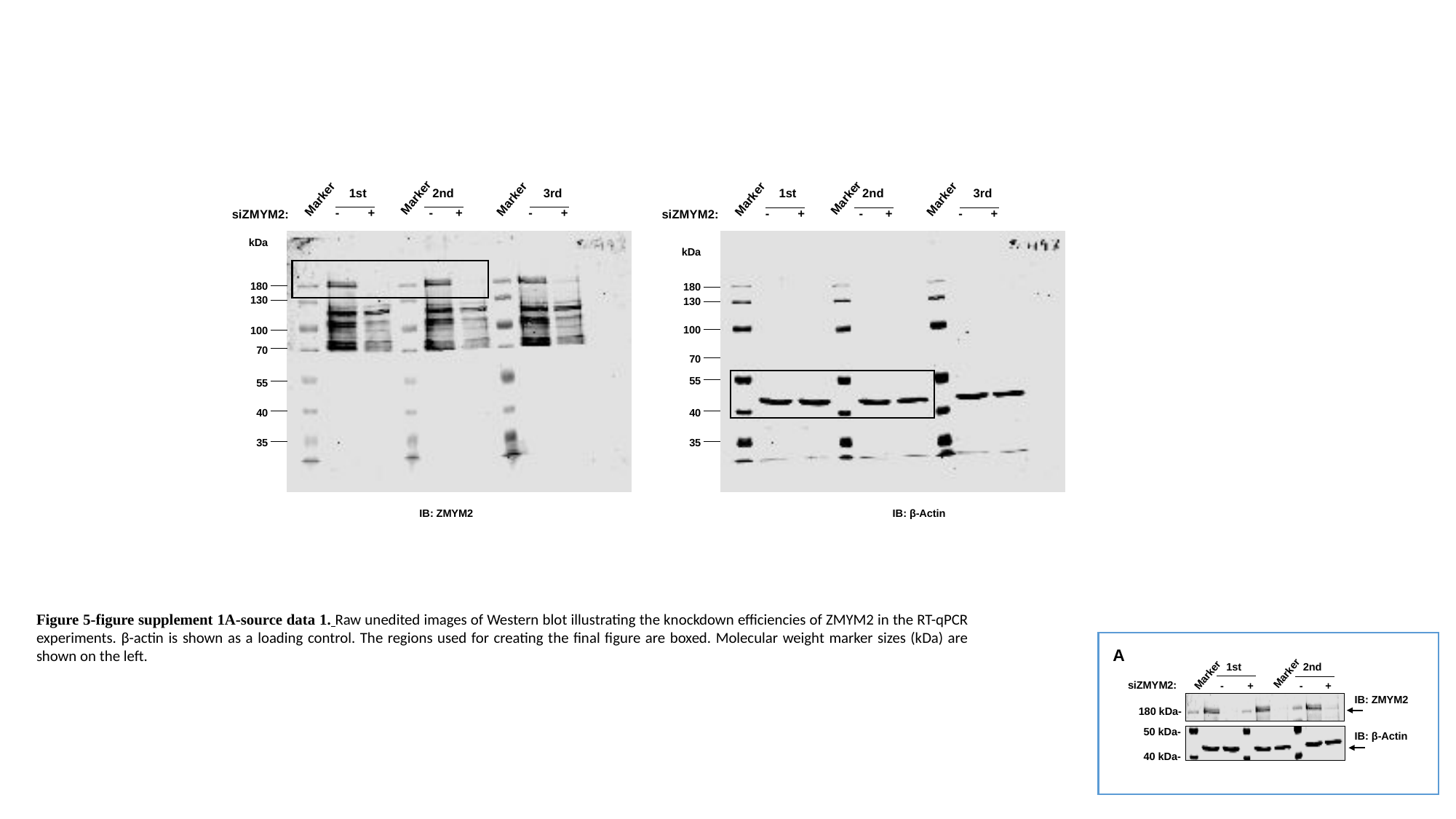

1st
2nd
3rd
1st
2nd
3rd
Marker
Marker
Marker
Marker
Marker
Marker
-
+
-
+
-
+
-
+
-
+
-
+
siZMYM2:
siZMYM2:
kDa
kDa
180
180
130
130
100
100
70
70
55
55
40
40
35
35
IB: ZMYM2
IB: β-Actin
Figure 5-figure supplement 1A-source data 1. Raw unedited images of Western blot illustrating the knockdown efficiencies of ZMYM2 in the RT-qPCR experiments. β-actin is shown as a loading control. The regions used for creating the final figure are boxed. Molecular weight marker sizes (kDa) are shown on the left.
A
1st
2nd
siZMYM2:
-
+
-
+
IB: ZMYM2
IB: β-Actin
Marker
Marker
180 kDa-
50 kDa-
40 kDa-
